# Integrated analysis of the chemical-material basis and molecular mechanisms for the classic herbal formula of Lily Bulb and Rehmannia Decoction in alleviating depression

**DOI:** 10.1186/s13020-021-00519-x

**Published:** 2021-10-21

**Authors:** Hongxiu Zhang, Xiaoyan Xue, Jin Pan, Xiaobin Song, Xing Chang, Qiancheng Mao, Yanting Lu, Haijun Zhao, Yuan Wang, Xiansu Chi, Shijun Wang, Ke Ma

**Affiliations:** 1 Shandong Co-Innovation Center of Classic TCM Formula, Shandong University of Traditional Chinese Medicine, No 4655, University Road, Changqing District, Jinan, 250355 Shandong People’s Republic of China; 2 Institute of Virology, Jinan Municipal Center for Disease Control and Prevention, Jinan, 250021 People’s Republic of China; 3 Department of Cardiology, Guang’anmen Hospital, Chinese Academy of Traditional Chinese Medicine, Beijing, 100053 People’s Republic of China; 4 Department of Brain Disease, Xiyuan Hospital, Chinese Academy of Traditional Chinese Medicine, Beijing, 100091 People’s Republic of China

**Keywords:** Lily bulb and rehmannia decoction, Bioactive ingredients, miRNA/mRNA regulatory network, GABAergic synapse, Neurotrophic signaling pathways, Homeostasis

## Abstract

**Background:**

Lily Bulb and Rehmannia Decoction (LBRD), is a traditional Chinese formula that has been shown to be safe and effective against depression; however, its material basis and pharmacological mechanisms remain unknown.

**Methods:**

Here, ultra-high performance liquid chromatography-quadrupole time-of-flight mass spectrometry (UHPLC-Q-TOF/MS) and high-performance liquid chromatography (HPLC) were used to identify the chemical spectrum and qualitatively identify the major active ingredients in the LBRD standard decoction, respectively. Subsequently, we assessed the behavior, neuronal function and morphology, neurotransmitter levels, hypothalamic–pituitary–adrenal (HPA)-axis associated hormones, inflammatory cytokine levels, and miRNA/mRNA expression alterations in an in vitro/vivo depression model treated by the LBRD standard decoction. Finally, miRNA/mRNA regulatory networks were created through bioinformatics analysis, followed by functional experiments to verify its role in LBRD standard decoction treatment.

**Results:**

A total of 32 prototype compounds were identified in the LBRD standard decoction, and the average quality of verbascoside in the fresh lily bulb decoction, fresh raw Rehmannia juice, and the LBRD standard decoction were 0.001264%, 0.002767%, and 0.009046% (w/w), respectively. Administration of the LBRD standard decoction ameliorated chronic unpredictable mild stress (CUMS)-induced depression-like phenotypes and protected PC12 cells against chronic corticosterone (CORT)-induced injury. The levels of neurotransmitter, cytokine, stress hormones and neuronal morphology were disrupted in the depression model, while LBRD standard decoction could work on these alterations. After LBRD standard decoction administration, four differentially expressed miRNAs, rno-miR-144-3p, rno-miR-495, rno-miR-34c-5p, and rno-miR-24-3p, and six differentially expressed mRNAs, *Calml4*, *Ntrk2*, *VGAT*, *Gad1*, *Nr1d1*, and *Bdnf* overlapped in the in vivo*/*vitro depression model. Among them, miR-144-3p directly mediated GABA synthesis and release by targeting *Gad1* and *VGAT*, and miR-495 negatively regulated BDNF expression. The LBRD standard decoction can reverse the above miRNA/mRNA network-mediated GABA and BDNF expression in the in vivo/vitro depression model.

**Conclusion:**

Collectively, the multi-components of the LBRD standard decoction altered a series of miRNAs in depression through mediating GABAergic synapse, circadian rhythm, and neurotrophic signaling pathway etc., thereby abolishing inhibitory/excitatory neurotransmitter deficits, recovering the pro-/anti-inflammatory cytokine levels and regulating the HPA-axis hormone secretion to achieve balance of the physiological function of the whole body.

**Supplementary Information:**

The online version contains supplementary material available at 10.1186/s13020-021-00519-x.

## Background

Depression is a frequent and highly heterogeneous mood disorder, and is a psychiatric condition characterized by the expression of anhedonia, despair, disturbed appetite or sleep, feeling of guilt and worthlessness, recurrent thoughts of death, and even suicidal tendencies [1]. Globally, more than 350 million people of all ages suffer from depression. Recent estimates from the China Mental Health Survey (CMHS) revealed that the lifetime prevalence of depressive disorder was reported to be 6.9% [2]. Despite its enormous impacts on the economy and human health, depression is still a highly pervasive with many co-morbidities for which the global scientific community has globally failed to provide the underlying genetic and environmental etiologies.

Currently, multiple clinical and animal studies lead to the formulation of several theories attempting to depict the pathogenesis of a depressive episode. Unfortunately, these hypotheses cannot fully clarify the perplexing processes of depression, and can only describe several pieces of this large puzzle [3]. It is particularly true considering the low treatment efficacy despite various types of targeted antidepressant drugs. This persistent incapacity for treating depression efficiently results in part from the heterogeneity of the disorder and the insufficient understanding of the functional and molecular mechanisms underlying its onset [4]. Hence, this is urgent need for immediate and effective treatments for depression with fewer adverse events, and complementary and alternative medicinal therapies, particularly traditional Chinese medicine (TCM), are widely tested for this purpose [5, 6]. TCM is a holistic treatment which emphasizes the integration of a variety of biological systems in the human body and the plants represent important raw materials for new compounds that warrant further investigation.

Lily Bulb and Rehmanniae Decoction (LBRD), recorded in the *Synopsis of Prescriptions of the Golden Chamber*, is a well-known classic TCM formula composed of lily bulb, raw Rehmannia root juice, and spring water based on the compatibility principle, and has the curative efficacy of nourishing yin and clearing heat, tonifying, and normalizing the heart and lung [7]. LBRD has been applied to clinical practice for treating "emotional diseases" in ancient China and neighboring countries or areas for thousands of years. Currently, it has been commonly used as an alternative therapy for treating depression, insomnia, perimenopausal syndrome, and lung diseases, among many others [8].

In previous studies, we have reported the original dosage and genuine producing area of lily, raw Rehmannia, and spring water, and defined the procedure of preparing the LBRD standard decoction [5]. Additionally, it has been shown to have therapeutic effects against stress-induced depression and is safe for use [9]. According the TCM theory, the formula exerts its effect through multi-components on various targets [10]. However, the complex chemical compositions and the scattered research results have blocked the systematic understanding of the material basis and compatibility principle of classic TCM formulas [11]. The chemical-material and quality control of the classical TCM agents are crucial to comprehensively improve its inheritance and innovative application [12]. The special active components and chemical spectrum in the LBRD standard decoction remains unclear. Therefore, we utilized ultra-high performance liquid chromatography quadrupole time of flight tandem mass spectrometry (UHPLC-Q-TOF/MS) to characterize the phytochemical-fingerprint and identify bioactive compounds of the LBRD standard decoction.

Nowadays, epigenetics, defined as heritable and reversible ways of regulating expression, has become an important subject in biological and medical sciences [13]. The overall concept and dynamic view of epigenetics is similar to the holistic view and therapy with syndrome differentiation of TCM theory [14]. The organic integration of TCM and epigenetics are an important approach to the future of integrating TCM and western medicine (WM), which could promote the development of both medicinal practices and would form a new research model of TCM. Previous studies have indicated that the imbalance of excitatory and inhibitory neurons in a depressive medial prefrontal cortex (mPFC) disrupts the coordination to the downstream neurons [15, 16]. The LBRD standard decoction could protect neuronal activity and abolish the inhibitory/excitatory neurotransmitters deficits via the multi-signaling pathway [9]. However, the pharmacological epigenetic mechanisms, especially from the perspective of miRNA/mRNA-mediated epigenetic regulation, by which the LBRD standard decoction alleviates pathological manifestations of depression, has not been studied.

In this study, we aimed to systematically reveal the material basis and pharmacological mechanism of action of the LBRD standard decoction in treating a chronic unpredictable mild stress (CUMS)-induced depressive behavior-like rat model and corticosterone (CORT)-induced rat adrenal pheochromocytoma (PC12) cell depression model. First, we used UHPLC-Q-TOF/MS to identify the chemical spectrum of single herbs and their combined formula. Simultaneously, high performance liquid chromatography (HPLC) was conducted to qualitatively determine major active ingredients constituting the formula and compare quality transitivity from single herbs to the LBRD standard decoction. Subsequently, we assessed changes in the behavior, neuronal function and morphology, neurotransmitter levels, the hypothalamic-pituitary-adrenocortical (HPA)-axis, inflammatory cytokine levels, and miRNA/mRNA expression in an in vitro*/*vivo depression model intervened with the LBRD standard decoction. Finally, miRNA/mRNA regulatory networks of the LBRD standard decoction against depression were created through bioinformatic analysis, followed by a series of experiments to verify its function in the antidepressant effect of the LBRD standard decoction. Through these comparisons and analyses, we expect to find its scientific basis of effective materials and the potential epigenetic mechanisms. This will impact the direction of future research, to provide a valuable reference for the secondary development and the new preparation design of classic prescriptions.

## Methods

### Animals and drug administration

Healthy, specific pathogen-free (SPF) male Sprague–Dawley (SD) rats (200 ± 20 g, 8 weeks) were obtained from Beijing Vital River Laboratory Animal Technology Co., Ltd (Beijing, China). All rats were raised individually in an aseptic flexible-plastic isolator under the following conditions: constant temperature of 22 ± 2 °C, relative humidity at 55 ± 5%, a 12 h light/dark cycle (lights on from 08:30–20:30), and free access to feed and water. All environmental factors such as light conditions, noise, or housing level were carefully controlled as these conditions may greatly influence stress actions in rats.

After a one-week acclimatization period, rats were randomly divided into four groups: control group, CUMS group, LBRD standard decoction-treated group and the fluoxetine-treated group. After a one-week of CUMS exposure, rats in the drug-treated groups were intragastric administrated with the LBRD standard decoction (150 g/kg) or fluoxetine (10 ml/kg) in a volume of 5 ml/kg for three weeks. Rats in the control and CUMS groups received an equivalent volume of saline water until the animals were sacrificed. Treatments were administered orally to the rats in each group 60 min before modeling. All animal experiments were carried out in accordance with the Guideline of National Institutes of Health, USA for the Care and Use of Laboratory Animals and approved by Animal Care and Use Committee of Shandong University of Traditional Chinese Medicine (SDUTCM201805311223).

### Preparation of the LBRD standard decoction

We have reported the genuinely producing area of the lily bulb, raw Rehmannia root juice and spring water, and also calculated the original doses of LBRD from ancient to modern volume [5]. The standard decoction of LBRD was made up of 400 g of fresh lily bulbs from Shennongjia (Hubei Province, China) and 400 g of fresh raw Rehmannia root from Jiaozuo (Henan Province, China), in a volume of 300 ml. The preparation process was as follows: (1) fresh lily bulbs (400 g) were washed and soaked in water for one night, decocted in 400 ml of spring water (Baotu Spring, Jinan, China) for 30 min, and herbs were removed to get 200 ml of decoction; (2) 400 g of fresh raw Rehmannia root were squeezed and filtered to yield about 200 ml of fresh raw Rehmannia root juice; and (3) 200 ml of fresh lily bulb decoction and 200 ml of fresh raw Rehmannia root were mixed and decocted with mild fire for 45 min to obtain a 300 ml mixture. We defined this decoction as the LBRD standard decoction.

### The CUMS paradigm and behavioral tests

The CUMS paradigm was performed with a minor modification as previously described and consisted of 12 stressors, social isolation, food and water deprivation, empty bottles, soiled cage, restraint space, circadian disturbance, white noise and space reduction, among others (Additional file 1: Table S1) [16–18]. These stressors were conducted between 08:00 and 22:00 (except 24 h of stressors) once daily in a randomized order for 28 consecutive days. Two days after building, behavioral tests were carried out to evaluate whether the rats expressed depression-like behavior. We used a battery of behavioral tests, including sucrose preference test (SPT), open field test (OFT), elevated plus maze test (EPMT), and forced swimming test (FST), performed in this particular order [19, 20]. Only one test was performed each day and approximately 24 h elapsed between tests. The behavioral tests were carried out between 09:00 and 20:00 in each group in a sound-proof behavioral facility and performed by a trained observer blinded to the type of treatment.

### UHPLC-Q-TOF/MS detection

The fresh lily bulb decoction, fresh raw Rehmannia juice, and the LBRD standard decoction were concentrated and dried under a vacuum environment. After being freeze-dried, the resulting dry extraction was dissolved in 2 ml of methanol/water solution (v/v, 1/4), and then mixed with a stock solution of mixed internal standards (L-2-chlorophenylalanine 0.3 mg/ml, lyso PC17:0 0.1 mg/ml, cholesterol-3,4-13C2 0.1 mg/ml, and glycocholic acid-13C1 0.1 mg/ml, each with a volume of 7.5 μl). After, the obtained mixture was centrifuged at 14,000 rpm for 10 min to obtain a 150 μl volume of the sample solutions. The supernatant was collected and filtered by a 0.22 μm nylon membrane prior to qualitative analysis.

UHPLC-Q-TOF/MS data-acquisition was conducted using a Triple TOF™ 6600 liquid chromatography high-resolution tandem mass spectrometer (AB SCIEX, Framingham, MA, USA) instrument equipped with a DuoSpray™ ion source. The chromatographic separation was done on an ACQUITY UPLC BEH C_18_ Column (2.1 mm × 100 mm, 1.7 μm; Waters, Milford, MA, USA). The mobile phase consisted of formic acid/water (0.1/100, v/v) (A) and acetonitrile (B) at a flow rate of 0.35 ml/min. The auto-sampler was maintained at 10℃, with an injection volume of 2.0 μl for all samples and the elution gradient of the lily bulb decoction, fresh raw Rehmannia root, and the LBRD standard decoction by UHPLC-Q-TOF/MS (Additional file 1: Table S2). The mass spectrometer detection was separately performed in a positive and negative ion mode using the electrospray ionization (ESI) ion source with use of mass parameters (Additional file 1: Table S3).

### HPLC qualitative analysis

The extract ingredient (verbascoside) from the LBRD standard decoction and its constituent herbs was analyzed with HPLC for quality control and bioactive compound analysis. The standards and samples (each 0.1 g) were prepared with 1 ml of acetonitrile, and filtered with 0.45 mm of organic membrane. The solution was performed on a Waters XBridge C18 reversed-phase column (2.1 mm × 100 mm, 3.5 µm) at a column temperature of 35 °C. Gradient elution of verbascoside was applied with phosphoric acid (0.5% A) and acetonitrile (B) as follows: 0–5 min, 5% B; 5–22 min, 15–25% B; 22–30 min, 25–45% B; 30–50 min, 45% B; 50–51 min, 45–15% B; and 51–60 min 15% B. The solvent flow rate was 1.0 ml/min and the injection volume was 10 µl with a 203 nm detection wavelength. Each sample was performed in triplicate. Data acquisition and processing were performed on an Agilent 6460 Triple Quadrupole mass spectrometer (Agilent Corporation, Santa Clara, CA, USA).

### Preparation of the LBRD standard decoction-containing serum

SD rats were randomly divided into two groups: the blank control group and the LBRD standard decoction group. Rats from the LBRD standard decoction group were administered with the LBRD standard decoction (150 g/kg/d) for three consecutive days, and the blank control group received an equal amount of saline by gavage. Abdominal aorta blood was collected from rats 2 h after administration of saline or the LBRD standard, and then centrifuged at low temperature for 20 min to avoid hemolysis, followed by heat-inaction in a water bath at 56 °C for 30 min. Finally, the obtained serum was sterilized through a 0.22 μm microporous membrane and stored at -80 °C for use after dispensing. All rats were anesthetized with sodium pentobarbital to minimize suffering and subsequently sacrificed by cervical dislocation after blood harvesting.

### Cell culture, treatments, and viability assay

PC12 cells (Kunming Institute of Zoology, Chinese Academy of Sciences, China) were seeded at a density of 1 × 10^5^/ml in high Dulbecco’s modified Eagle’s medium (DMEM, Corning, NY, USA) supplemented with 10% heat-inactivated fetal bovine serum and 1% penicillin–streptomycin and cultured at 37 °C under 5% CO_2_ for 24 h. The culture media was replaced every other day, and cells in the exponential phase of growth were applied for all experiments. Plated PC12 cells were exposed to 200 and 400 μM of CORT with a purity of 95% (Sigma-Aldrich, St Louis, MO, USA) for 24 h and then treated with different concentrations of the LBRD standard decoction-containing serum (5%, 10%, and 20%). The CCK-8 assay was performed to evaluate cell viability. PC12 cells were seeded in 96-well plates and cultured, then treated with the CCK-8 solution and incubated at 37 °C for 1 h. The optimal density of each well was measured at 450 nm with a microplate reader. Cell viability was expressed as a percentage of control cells and the assay was repeated in triplicate.

### Histopathological and biochemical indicator analysis

SD rats were anesthetized via sodium pentobarbital, and physiological saline was replaced with a slow drip of 4% polyformaldehyde for 30 min after rapid perfusion via the heart. The brain was than removed and post-fixed in 4% formaldehyde solution overnight at 4 °C. After being dehydrated and embedded in paraffin, the mPFC brain tissues were cut into 5 μm-thick coronal sections. The paraffin sections were deparaffinized in xylene and rehydrated in a series of diluted ethanol and double-distilled water, and then stained with 1% hematoxylin and eosin. The slides were subsequently dehydrated with ethanol at gradient concentrations. Five non-overlapping fields of view were randomly selected in each slice under a light microscope (Olympus Optical Co Ltd; Tokyo, Japan). The integral optical density was determined using the Image-Pro Plus 6.0 system for semi-quantitative analysis.

The expression levels of neurotransmitters, cytokines, and hormones from the mPFC of rats and cell serum were measured using the enzyme linked immunosorbent assay (ELISA). All the kits were purchased from Beyotime Biotechnology Co., Ltd., Shanghai, China and the assay was performed according to manufacturer’s instructions. Each experiment was conducted in triplicate.

### RNA-sequencing and bioinformatic analysis

High-throughput sequencing of transcriptional profiles were performed by Shenzhen BGI Gene Science and Technology Co., Ltd. In brief, total RNA was extracted from the rat mPFC tissues and PC12 cells with the RNAiso Plus kit (Takara, Dalian, China) and was measured with ultraviolence absorbance to assess its purity and quality. The samples with a concentration of > 200 ng/μl and a A260/A230 ratio of > 2 were chosen for miRNA and mRNA expression profile sequencing using the GISEQ-500 platform (BGI., Shenzhen, China). The average read length of two libraries was about 100 bp (pair-end) and 50 bp (single-end), respectively.

The raw sequencing reads of mRNA and miRNA expression from the mirDeep2 outputs were used for analysis. After filtering, high-quality clean reads were matched to the rat genome reference sequence (GCF_000001895.5_Rnor_6.0) and miRNA sequence database (miRBase) by using TopHat v1.0.12 which incorporated Bowtie v0.11.3 software to perform the alignments. Uniquely localized reads were used to calculate read number and the reads per kilobase of exon model per million mapped reads value of each gene and the sole reads uniquely aligned to the genes were used to quantify the mRNA and miRNA expression levels. The voomlimma R package method equipped with DESeq2 package of bioconductor was used to screen the differentially expressed mRNAs and miRNAs [21, 22], which were filtered with a false discovery rate (FDR) of ≤ 0.05 and a fold change (FC) of ≥ 1.5, and were selected for functional enrichment analysis and target gene predictions, respectively [15, 17].

To identify potential miRNA-regulated target genes, the datasets of differentially expressed miRNAs and transcripts were integrated. We set the following criteria for the potential targets: (1) the target mRNAs should be predicted in all of these software TargetScan (http://www.targetscan.org), Rnahybrid (http://bibiserv.techfak.uni-bielefeld.de/rnahybrid/RNAhybrid), and Miranda (http://www.mirbase.org), where the principle for the prediction of miRNA targets includes the matched seeds, accessible sites, free energy and conservation; (2) the compliant miRNA target predictions were overlapped to those of differentially expressed mRNAs from transcriptome sequencing; and (3) the differentially expressed miRNAs and the overlapped mRNAs should be simultaneously and reversely changed in different experiment groups. Interactive networks from the differentially expressed miRNAs and the simultaneously expressed target mRNAs were visualized using the Cytoscape software (San Diego, CA, USA).

### Quantitative RT-PCR and Western blot analyses

Total RNAs from rat mPFC tissues and PC12 cells were extracted using Trizol (Thermo Fisher, Waltham, MA, USA) following the manufacturer's instructions. Thereafter, the cDNA was generated with the use of a reverse-transcription kit (Takara, Dalian, China) and amplified using the Power SYBR-Green Premix (Takara, Dalian, China) by the ABI 7500 System (Applied Biosystems, Foster City, CA, USA). The amplification levels of the mRNAs and miRNAs relative to controls were computed based on the 2-ΔΔCt method. GAPDH and U6 served as the internal controls for the normalization of qRT-PCR data. Each sample was prepared at least three times.

For western blot analysis, total protein from mPFC brian tissues and cultured PC12 cells were first lysed using the M-PER Mammalian Protein Extraction Reagent (Thermo Fisher Scientific, CA, USA). The equivalent amount of protein was than fractionated on a 12.5% sodium dodecyl sulfate–polyacrylamide gel electrophoresis (SDS-PAGE, Beyotime, Shanghai, China) and electro-transferred onto nitrocellulose membranes (Millipore, Darmstadt, Germany). Following blocking with 5% non-fat milk for 1 h at room temperature, the samples were subsequently probed with each candidate antibody at 4 °C for 12 h. Then the membranes were incubated with fluorescently labeled secondary antibodies for 2 h. Finally, the level of protein expression was visualized and evaluated using the Gel Doc XR + automatic gel imaging system (Bio-Rad, CA, USA) and Image J software [23].

### Dual luciferase and RNA immunoprecipitation (RIP)

The wild type or mutant containing the putative binding sites of candidate miRNAs were amplified and cloned into the pGL4-control luciferase reporter vectors (Promega Corporation, Madison, WI, USA). Subsequently, the HEK293T cells were co-transfected with the reporter plasmid and candidate miRNA mimics or miR-NC using Lipofectamine 2000 (Invitrogen). After 48 h, the luciferase activity was analyzed by a dual-luciferase reporter assay system (Promega Corporation) and normalized to Renilla luciferase activity. RNA immunoprecipitation (RIP) experiments were carried out with the EZ-Magna RIP kit (Millipore, Billerica, MA, USA) according to the manufacturer’s protocol. PC12 cells were lysed in complete RIP lysis buffer, and the cell extract was incubated with protein A/G agarose beads conjugated with antibody Bsg or control IgG for 2 h at 4 °C. Beads were washed and incubated with Proteinase K to remove proteins. Finally, purified RNA was subjected to qRT-PCR analysis. Each sample was measured in triplicate to get the average.

### Statistical analyses

All experiments were repeated at least in triplicate prior to statistical analysis and the results were expressed as mean ± standard error of mean (SEM). Differences between two groups were compared by Student’s *t*-test, while the comparison between three or more groups were performed through one-way analysis of variance (ANOVA) followed by Dunnett’s test to evaluate the variance of multiple groups. Spearman’s correlations were used to assess the correlations among the expression of miRNAs and target mRNAs. Statistical significance was set at *P* < 0.05.

## Results

### Identification of the LBRD standard decoction constituents by UHPLC-Q-TOF/MS

**Fig. 1:**
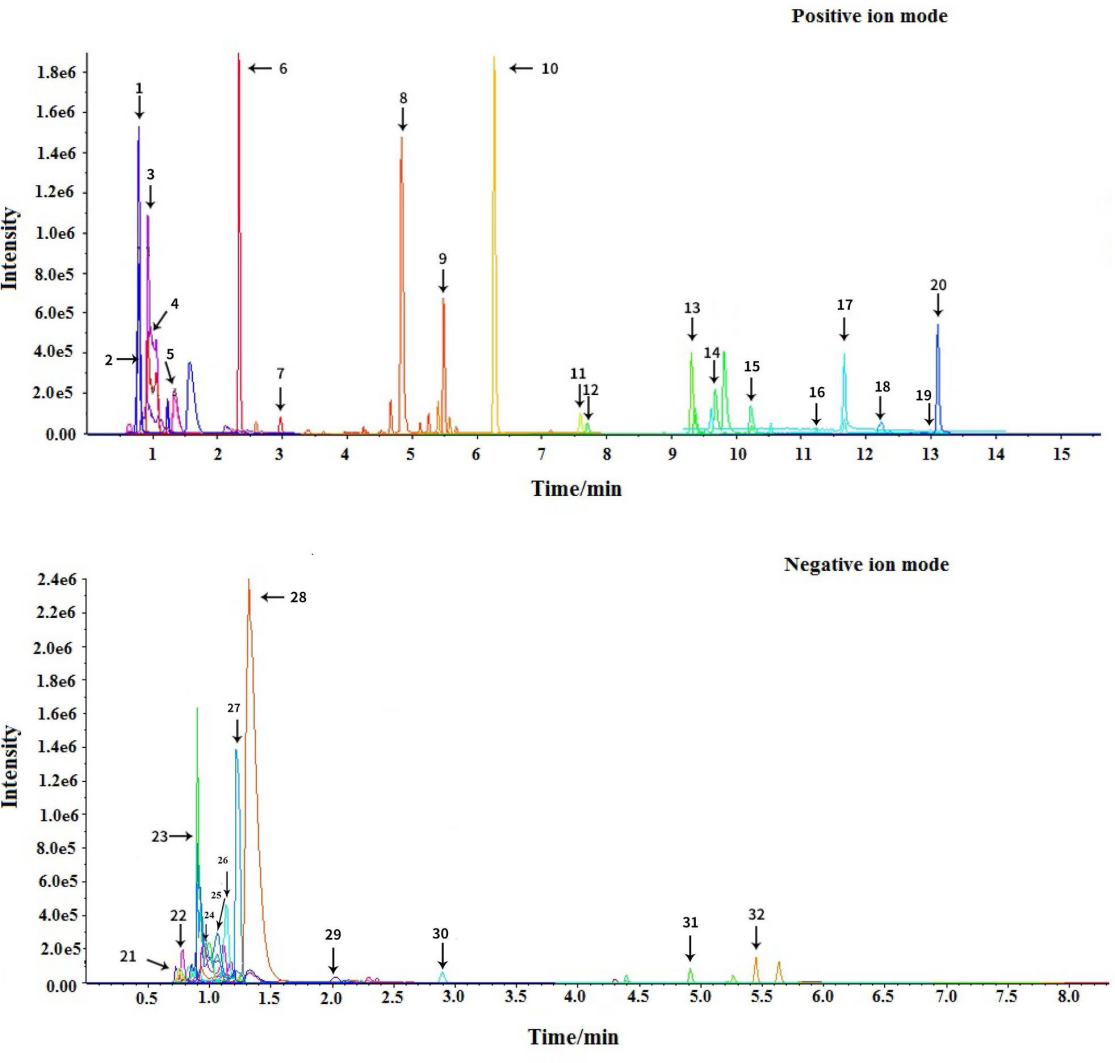
Total ion chromatogram of LBRD standard decoction in positive and negative ion mode using UHPLC-Q-TOF/MS. The samples were analyzed in positive (**A**) and negative (**B**) modes, ranging from m/z 50–1500. The UHPLC-Q-TOF/MS information of all the compounds were obtained and the data were processed using Thermo Xcalibur 3.0 software. Elements in use (**C** 0–40, H 0–80, O 0–30, and Na 0–1) and mass tolerance (< 5 ppm for diterpenes, lactones, and flavonoids, and < 10 ppm for phenolic acids) were set accordingly to narrow down the options for molecular analysis

The response values of different chemical compositions differ in different modes. Hence, we undertook UPLC-QTOF-MS/MS in positive and negative ionization modes. The UHPLC-Q-TOF/MS conditions were first optimized systematically to obtain the good chromatographic separation and appropriate ionization. The total ion current (TIC) chromatograms of the LBRD standard decoction are shown in Fig. 1. A total of 32 prototype compounds were identified in the LBRD standard decoction, and the herbs they were derived from could be determined by comparing the retention time and MSn data from fresh lily bulb and raw Rehmannia root (Additional file 1: Figure S1) as listed in Table 1. Among them, 29 ingredients were primarily from fresh lily bulb, 28 from fresh raw Rehmannia juice, and 25 presented in both herbs. There are four and three compounds individually attributed to fresh lily bulb and fresh Rehmannia juice, respectively. The main ingredients in the LBRD standard decoction were classified as polysaccharides, phenolic acid glycerides, glycosides, and iridoid glycosides.

**Table 1:** Characterization of the chemical constituents in Lily Bulb and Rehmannia Decoction (LBRD) standard decoction by UPLC-QTOF-MS/MS

Number	Component	Model (±)	Retention time (min)	Formula	M/Z	Resource
1	L-4-Hydroxyglutamate semialdehyde	+	0.7774	C5H9NO4	148.06	Both
2	Verbascoside	+	0.8489	C29H36O15	624.59	Both
3	L-2-Aminoadipic acid	+	0.9214	C6H11NO4	162.08	Both
4	6-Oxopiperidine-2-carboxylic acid	+	0.9214	C6H9NO3	144.07	Fresh lily bulb decoction
5	2,5-Furandicarboxylic acid	+	1.315	C6H4O5	139	Both
6	Vidarabine	+	2.2965	C10H13N5O4	268.1	Both
7	Aminoadipic acid	+	2.9324	C6H11NO4	144.07	Fresh lily bulb decoction
8	Coumarin	+	4.8195	C9H6O2	147.04	Fresh lily bulb decoction
9	8-tridecynoic acid	+	5.3891	C13H22O2	193.16	Both
10	Triethyl phosphate	+	6.2625	C6H15O4P	183.08	Both
11	Myristic acid	+	7.5879	C14H28O2	246.24	Both
12	( +)-15S-hydroxy-hexadecanoic acid	+	7.6919	C16H32O3	290.27	Both
13	17-hydroxy stearic acid	+	9.3046	C18H36O3	318.3	Both
14	14:0(5Me[R],9Me[R],13Me)	+	9.6715	C17H34O2	288.29	Both
15	Stearic acid	+	10.2174	C18H36O2	302.31	Both
16	2E,6Z,8Z,12E-hexadecatetraenoc acid	+	11.2287	C16H24O2	249.18	Both
17	Monoethylhexyl phthalic acid	+	11.6635	C16H22O4	301.14	Both
18	Polidocanol	+	12.2376	C30H62O10	600.47	Both
19	1-O-(2R-hydroxy-hexadecyl)-sn-glycerol	+	12.9221	C19H40O4	333.3	Both
20	Oleamide	+	13.1112	C18H35NO	304.26	Both
21	2-Anthramine	−	0.7189	C14H11N	385.17	Both
22	N-Methyl-D-aspartic acid	−	0.766	C5H9NO4	146.05	Both
23	Kojibiose	−	0.8957	C5H9NO4	341.11	Both
24	Maltotriose	−	0.9073	C18H32O16	549.17	Both
25	Maltotetraose	−	1.0472	C24H42O21	711.22	Both
26	Osmundalactone	−	1.1405	C6H8O3	173.05	Both
27	Ikarisoside D	−	1.2228	C28H30O11	587.18	Both
28	5-methoxy-4-methylbenzene-1,3-diol	−	1.3967	C8H10O3	199.06	Fresh raw Rehmannia juice
29	Catalpol	−	1.9963	C15H22O10	407.12	Fresh raw Rehmannia juice
30	Estradiol-17beta 3-sulfate	−	2.89	C18H24O5S	397.13	Both
31	Dihydroartemisinin	−	4.9027	C15H24O5	283.15	Fresh raw Rehmannia juice
32	Diosbulbinoside F	−	5.41	C26H34O12	519.19	Fresh lily bulb decoction

We next used HPLC to perform qualitative determination of verbascoside which was present in two herbs and was an effective antidepressant agent, and then compared quality transitivity from the single herb to the LBRD standard decoction. The results from HPLC chromatograms showed that verbascoside in the LBRD standard decoction and its constituent single herb matched the corresponding peaks of standard by the same elution system (Additional file 1: Figure S2). Quantitative analysis displayed the average quality of verbascoside in the fresh lily bulb decoction, fresh raw Rehmannia juice, and the LBRD standard decoction were 0.001264% (w/w), 0.002767% (w/w), and 0.009046% (w/w), respectively (Additional file 1: Table S4). These results were in line with the standard qualities of the lily bulb and Rehmannia root in the *Pharmacopoeia of People's Republic of China* (2015 Edition). According to quality transitivity, the verbascoside concentrations in the fresh lily bulb decoction, the raw Rehmannia juice, and LBRD standard decoction increased from low to high. Thus, verbascoside might serve as the quality marker for the LBRD standard decoction.

### LBRD standard decoction attenuated the depressive symptoms in rats exposed to CUMS

**Fig. 2:**
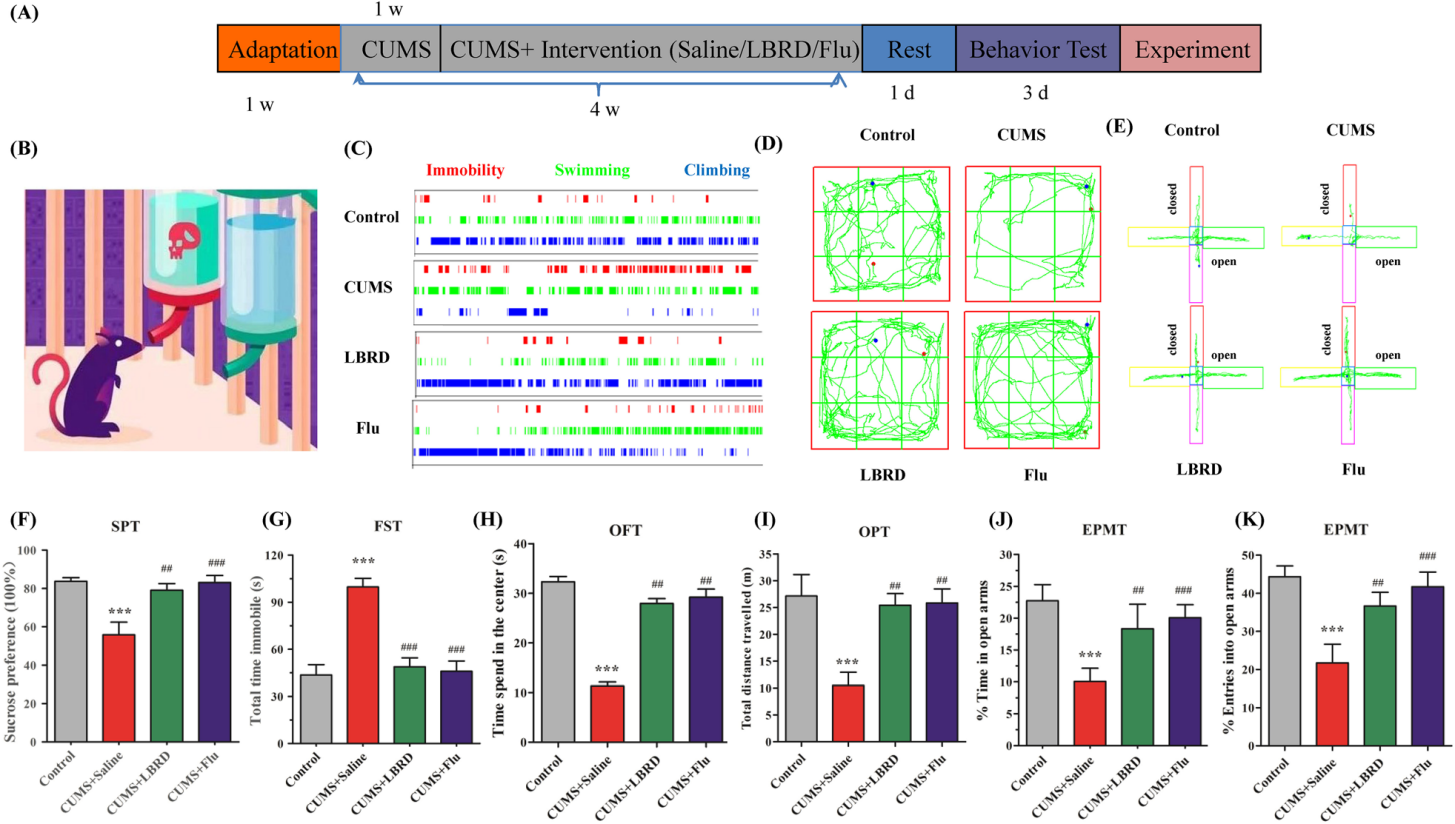
LBRD standard decoction attenuates the depressive and anxiety symptoms in chronic stress exposed rats. **A** The timeline for chronic stress induction, LBRD standard decoction treatment, and behavior evaluation. **B** Schematic diagram of the sugar water preference test. **C** Representation of the time spent in the status of immobility, swimming, and climbing of rats in FST. **D** The movement trails of rats in OFT. **E** Representative diagram in the probe trial in EPMT. **F** The percent of sucrose consumption/total water in the SPT. **G** Total time spent immobile in FST. **H** Active time of rats in the center of OFT. **I** Total distance traveled in OFT. **J** Percentage of active time in the open arms of EPMT. **K** Percentage of entries into the open arms of EPMT. All data are presented as mean ± SEM (n = 12 per group) and differences between two groups were compared by Student’s *t*-test. ^***^P < 0.001, compared to control group; ^##^*P* < 0.01, ^###^*P* < 0.001 compared to CUMS + Saline group. CUMS + Saline: CUMS + Saline group, CUMS + Flu: CUMS + fluoxetine group, CUMS + LBRD: CUMS + Lily Bulb and Rehmannia Decoction group

To determine whether LBRD standard decoction intervention has an effect on the depressive symptoms and anxiety-like behavior in the CUMS rats, several behavioral tests were carried out, including SPT, TST, OFT, and EPMT (Fig. 2A). The SPT and FST were used to assess depressive-like behavior, while the OFT and EPMT were used to assess locomotor ability and anxiety-like behavior. The SPT was often used to measure depression-like behavior in rats by the evaluation of hedonic state or the ability to gain pleasure (Fig. 2B). In the SPT, we found that the sucrose consumption was reduced in the case of the CUMS-treated rats compared to the control rats (*P* < 0.001); whereas, the LBRD standard decoction and fluoxetine treatment for 21 consecutive days with 150 g/kg and 10 mg/kg, respectively, notably increased the sucrose consumption than that in the depression group (*P* < 0.01, Fig. 2F). FST was another classical behavior test for depression-like behavior by recording the time spent in immobility, swimming, and climbing (Fig. 2C). In the FST, the immobility time was significantly increased in rats of the CUMS group after 4 weeks of chronic stress procedure. In contrast, the duration of immobility in the CUMS + LBRD standard decoction group was half less than that of the CUMS group (Fig. 2G). The above-mentioned finding confirmed depression-like behavior in the CUMS rats and revealed that LBRD standard decoction administration can ameliorate this behavior.

The total time in the central area (Fig. 2H) by the rats in the CUMS group, as well as total distance of motion in 4 min (Fig. 2I), was significantly reduced compared with that in the control group. However, compared with the CUMS group, those behaviors were ameliorated after administration with the CUMS + LBRD standard decoction and fluoxetine (both *P* < 0.01). In the EPMT, the total active time and number of entries into open arms in the LBRD standard decoction treatment group were comparable to those in the control group (Fig. 2J, K). These results indicate the LBRD standard decoction may have therapeutic effects for treating anxiety-like behavior.

### LBRD standard decoction ameliorated neurotransmitter, inflammatory cytokine, and circulating stress hormone levels

**Fig. 3:**
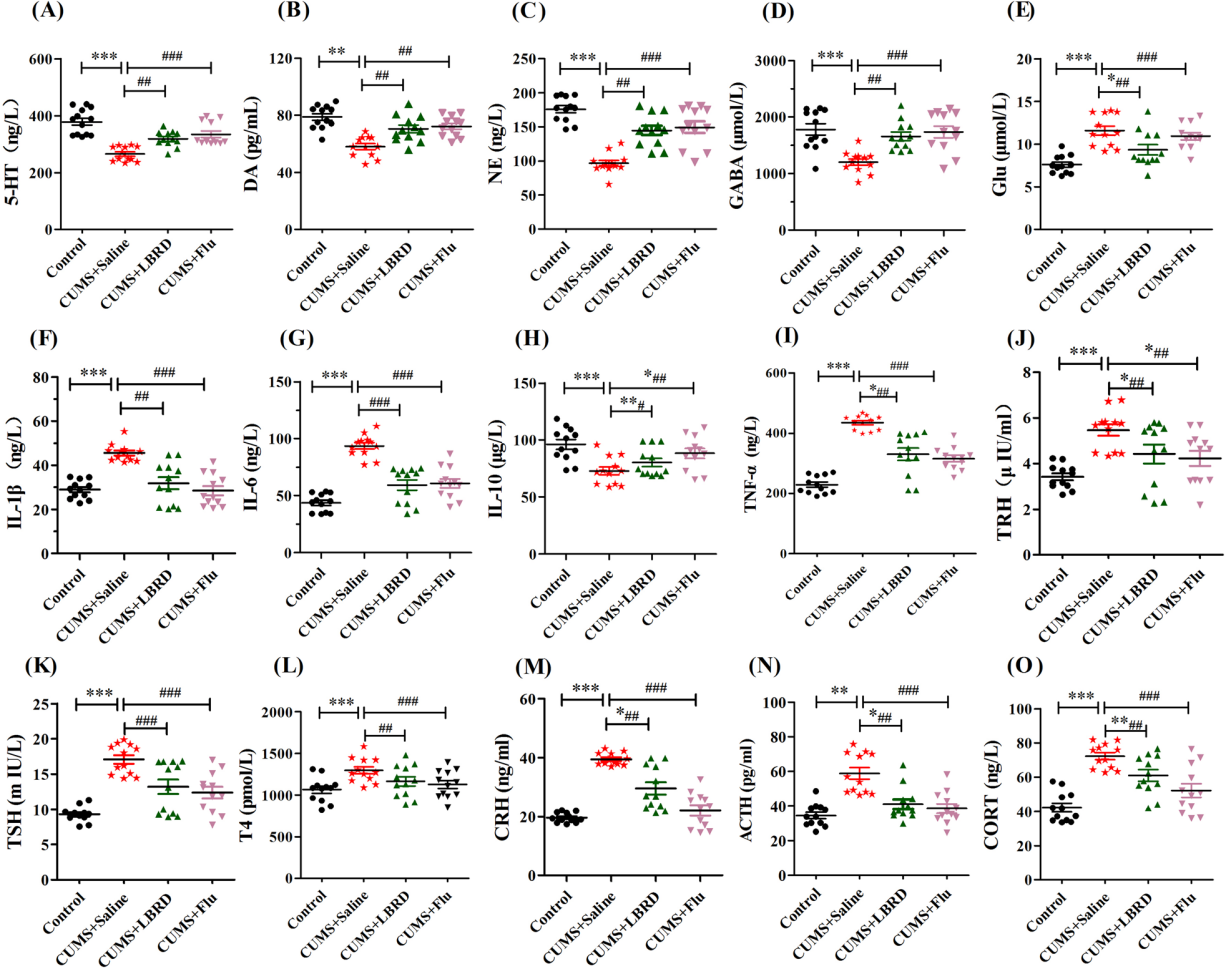
LBRD standard decoction ameliorates neurotransmitter, inflammatory cytokine and circulating stress hormone levels in the mPFC tissue and serum of the rats exposed to CUMS. LBRD standard decoction or fluoxetine treatment significantly increased the level of 5-HT (**A**), NE (**B**), DA (**C**), GABA (**D**), IL-10 (**H**), and decreased Glu (**E**), IL-1β (**F**), IL-6 (**G**), TNF-α (**I**), TRH (**J**), TSH (**K**), T4 (**L**), CRH (**M**), ACTH (**N**), and CORT (**O**). Data are expressed as mean ± SEM (n = 12 per/group) and differences between two groups were compared by Student’s *t*-test. ^*^P < 0.01, ^**^P < 0.01, ^***^P < 0.001, compared to control group; ^#^*P* < 0.05, ^##^*P* < 0.01, ^###^*P* < 0.001, compared to CUMS + Saline group. CUMS + Saline: CUMS + Saline group, CUMS + Flu: CUMS + fluoxetine group, CUMS + LBRD: CUMS + Lily Bulb and Rehmannia Decoction group

Next, we investigated the effects of LBRD standard decoction administration on the level of neurotransmitter and inflammatory cytokine in the mPFC tissues, and circulating stress hormone in the serum from CUMS-induced depression-like behavior rats by ELISA. Compared with the control group, the level of monoamine neurotransmitters such as 5-hydroxytryptamine (5-HT), dopamine (DA), norepinephrine (NE), and gamma-aminobutyric acid (GABA) from mPFC tissue of rats in the CUMS group displayed a significant decrease, but the level of glutamate (Glu) increased. However, 3 weeks after the LBRD standard decoction or fluoxetine intervention, this reduction could be effectively restored (Fig. 3A-E). In terms of pro-inflammatory cytokines, the levels of interleukin (IL)-1β, IL-6, and tumor necrosis factor-α (TNF-α) in the LBRD standard decoction treated-group, were significantly decreased than those in the CUMS group with statistical difference (Fig. 3F, G and I, both *P* < 0.01). On the contrary, the expression level of anti-inflammatory cytokine, IL-10, was notably increased in the LBRD standard decoction treatment group (Fig. 3H, both *P* < 0.01). On circulating stress hormone levels, the plasma levels of the HPA axis hormones, such as thyroid stimulating hormone (TSH), thyroxine (T4), corticotropin-releasing hormone (CRH), adrenocorticotropic hormone (ACTH), and CORT significantly increased in depression group compared to those in the control group, while the LBRD standard decoction treatment significantly attenuated this increase (Fig. 3K–O, both *P* < 0.01). Collectively, this data indicated that LBRD standard decoction treatment ameliorated depressive symptoms by abolishing inhibitory/excitatory neurotransmitter deficits, recovering the balance of pro/anti-inflammatory cytokine, and regulating HPA axis hormone levels.

### Effects of LBRD standard decoction on histological alterations in mPFC tissue from CUMS-induced depression-like behavior rats

**Fig. 4:**
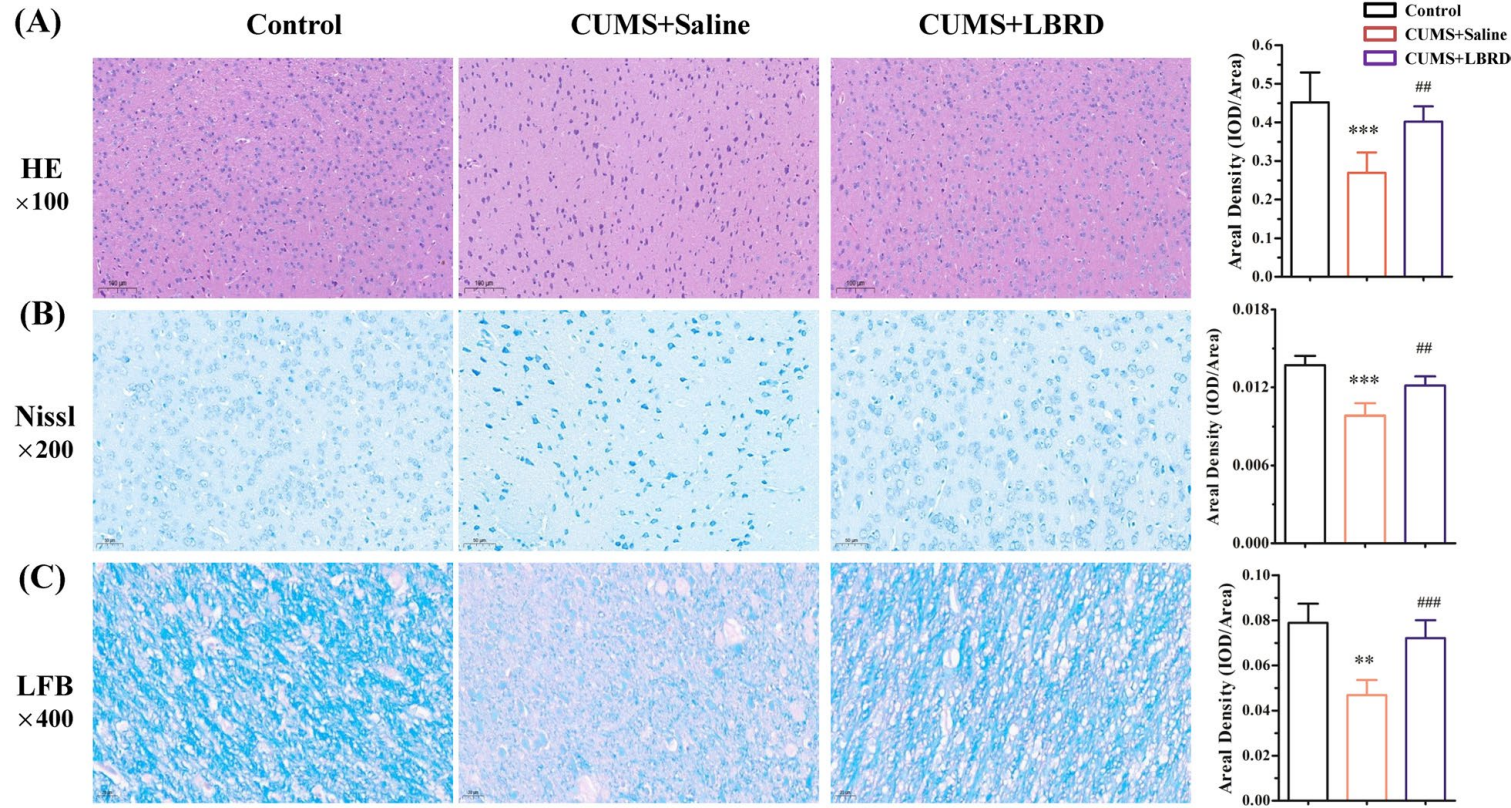
LBRD standard decoction has a protective effect on neuronal morphology in CUMS-induced depression like behavior rats. **A** Morphological changes of prefrontal cortex nerve cells in each experimental group (H&E, ×100). **B** Morphological changes of prefrontal cortex neuronal cells in each experimental group (Nissl, ×200). **C** Morphological changes of prefrontal cortex myelinated nerve fibers in each experimental group (LFB, ×400). Five non-overlapping fields of view were randomly selected in each slice under a light microscope. The integral optical density was determined using the Image-Pro Plus 6.0 system for semi-quantitative analysis. Data are expressed as mean ± SEM (n = 9–12 per/group) and differences between two groups were compared by Student’s *t*-test. ^**^P < 0.01, ^***^P < 0.001, compared to control group; ^##^*P* < 0.01, ^###^*P* < 0.001, compared to CUMS + Saline group. CUMS + Saline: CUMS + Saline group, CUMS + LBRD: CUMS + Lily Bulb and Rehmannia Decoction group

Hematoxylin & eosin staining was performed to observe the morphological changes in the neurons of the rat mPFC tissue in the different groups. The results revealed that prefrontal cortex nerve cells of rats exposed to CUMS for 4 weeks showed an irregular arrangement and abnormal aggregation compared to rats in the control, and this condition was effectively ameliorated in the LBRD standard decoction treatment group (Fig. 4A). Furthermore, the data from Nissl staining showed that large numbers of prefrontal cortex neuronal cells from the depressive group exhibited karyopyknosis, interstitial edema, neuronal loss, ruptured nuclear membrane, and disappeared nucleolus, in comparison with the control group. After drug administration, LBRD standard decoction markedly alleviated pyknosis and increased the number of Nissl bodies in the mPFC tissues (Fig. 4B). Quantitative image analysis of luxol fast blue (LFB) stained sections was undertaken to assess the pathological alterations of myelin sheaths. The arrangement of prefrontal cortex myelinated nerve fibers in the model group was disordered, and there was loose neuropil with vacuolar disintegration. Additionally, the ratio of the injured myelinated nerve fibers was significantly higher than in the control group. However, in the groups were treated with the LBRD standard decoction, the extent of damage was significantly alleviated, and the myelinated nerve fibers were also markedly well-arranged (Fig. 4C). These observations suggested that there were pathological alterations in mPFC tissues in depression, and the LBRD standard decoction has a protective effect on neuronal morphology.

### Identification of miRNA/mRNA regulatory network for LBRD standard decoction treatment of depression

**Fig. 5:**
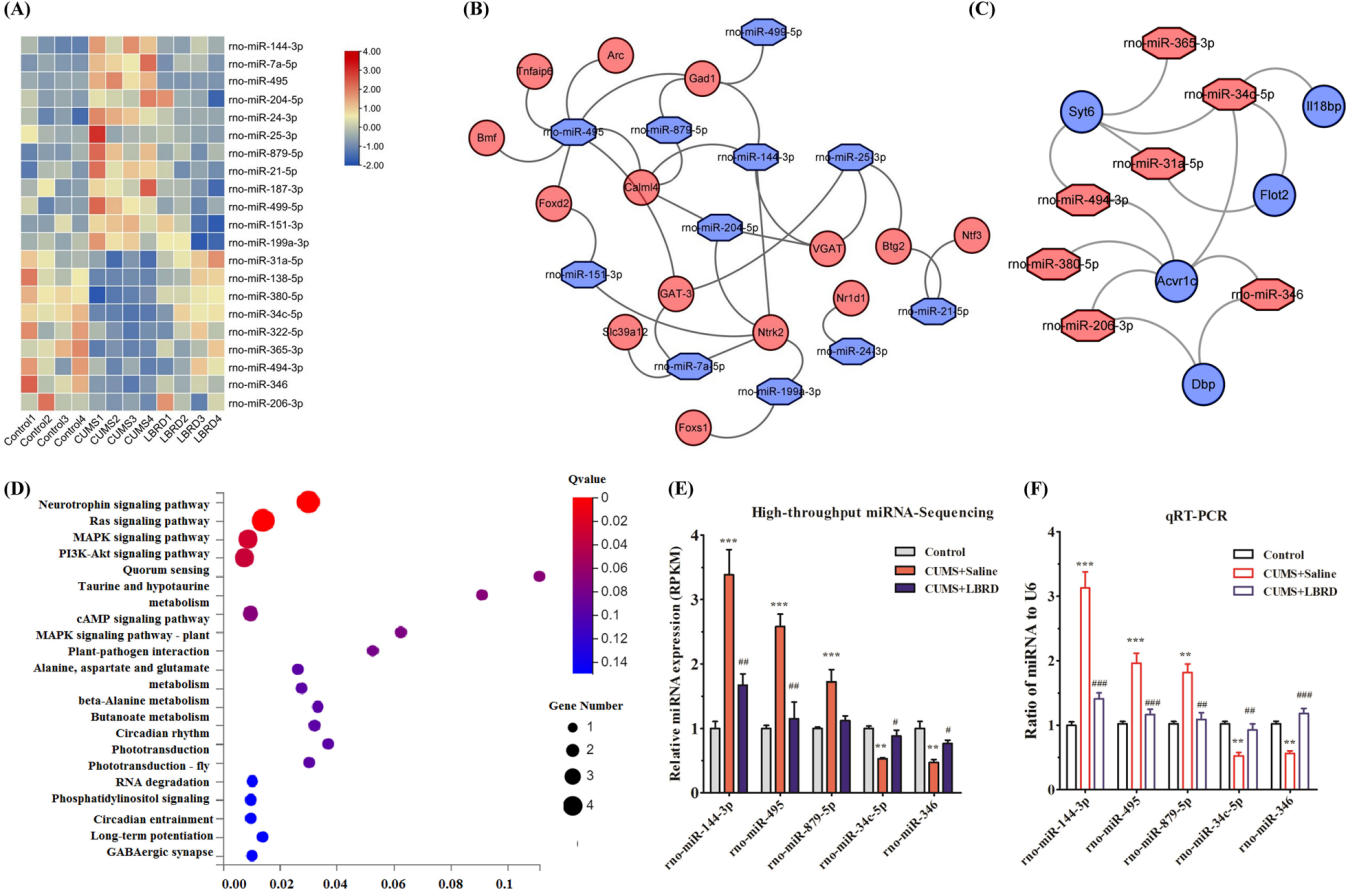
miRNA/mRNA regulatory network of LBRD standard decoction for CUMS-induced rats depression treatment. **A** Heat map from deep-sequencing depicting the most significant differentially expressed miRNAs from mPFC tissues among experimental groups. **B** Interactive network of down-regulated miRNAs targeting up-regulated mRNAs for the antidepressant effect of the LBRD standard decoction. **C** Interactive network of up-regulated miRNAs targeting down-regulated mRNAs for the antidepressant effect of the LBRD standard decoction. Blue represents down-regulated miRNAs and up-regulated mRNAs from the LBRD standard decoction compared to the CUMS group, as well as the CUMS group compared to the control. Red represents up-regulated miRNAs or down-regulated mRNAs. **D** KEGG pathway of those predicted target mRNAs overlapping with DEGs in the transcriptome in mPFC tissues derived from CUMS-induced rats with depression treated with the LBRD standard decoction. CUMS-induced rat depression model. **E** The relative level of rno-miR-144-3p, rno-miR-495, rno-miR-879-5p, rno-miR-34c-5p, and rno-miR-346 from mPFC tissues (n = 4 per/group), which were analyzed by high-throughput miRNA sequencing. **F** qRT-PCR was used to analyze the relative values of rno-miR-144-3p, rno-miR-495, rno-miR-879-5p, rno-miR-34c-5p, and rno-miR-346 (n = 8–12 per/group), including the cases used for high-throughput sequencing. U6 was set as the internal control. Differences between two groups were compared by Student’s *t*-test. **P < 0.01, ***P < 0.001, compared to control group; ^##^*P* < 0.01, ^###^*P* < 0.001, compared to CUMS + Saline group. CUMS + Saline: CUMS + Saline group, CUMS + LBRD: CUMS + Lily Bulb and Rehmannia Decoction group

We analyzed the transcriptional landscape of mPFC tissues to determine the epigenetic mechanisms underlying the LBRD standard decoction against depression by high throughput sequencing. In each of CUMS/control and LBRD/CUMS comparisons, differentially expressed miRNAs with FC ≥ 1.5 and FDR ≤ 0.05 with the reverse change, were considered as candidate miRNAs for further analysis. Based on these criteria, there were 21 differentially expressed miRNAs in CUMS/control and LBRD/CUMS (Table 2). Among them, 12 miRNAs such as rno-miR-144-3p, rno-miR-495, rno-miR-204-5p, rno-miR-879-5p, etc., were significantly down-regulated in the mPFC tissues from the LBRD standard decoction treated group, as well as nine different miRNAs (miR-31a-5p, miR-138-5p, miR-380-5p, miR-34c-5p, miR-322-5p, miR-365-3p, miR-494-3p, miR-346 and miR-206-3p), were up-regulated compared to those from the model groups (Fig. 5A).

**Table 2:** miRNAs with quantitative change over 1.5 folds and their characteristics in rats mFPC tissues

miRNA	Accession no	Control Means	CUMS Means	LBRD Means	Fold Change CUMS/Control	Fold Change LBRD /CUMS	Chromosomal location (Rat)	Seed sequence
rno-miR-144-3p	MIMAT0000850	19.55	57.73	28.54	2.95	0.49	chr10: 65,291,365–65,291,447 [−]	5′-ACAGUAU-3′
rno-miR-7a-5p	MIMAT0000606	376.61	995.99	450.90	2.64	0.45	chr17: 6,675,016–6,675,112 [+]	5′-GGAAGAC-3′
rno-miR-495	MIMAT0005320	589.33	1520.44	472.28	2.58	0.31	chr6: 133,868,288–133,868,367 [+]	5′-AACAAAC-3′
rno-miR-204-5p	MIMAT0000877	249.69	458.77	296.66	1.84	0.65	chr1: 240,403,000–240,403,109 [+]	5′-UCCCUUU-3′
rno-miR-24-3p	MIMAT0000794	2808.93	5124.21	3378.83	1.82	0.66	chr17: 823,968–824,035 [+]	5′-GGCUCAG-3′
rno-miR-25-3p	MIMAT0000795	92.61	161.63	60.95	1.75	0.38	chr12: 19,307,340–19,307,423 [−]	5′-AUUGCAC-3′
rno-miR-879-5p	MIMAT0005287	1.64	2.83	1.84	1.72	0.65	chr4: 21,673,635–21,673,710 [−]	5′-GAGGCUU-3′
rno-miR-21-5p	MIMAT0000790	226.30	388.76	225.74	1.72	0.58	chr10: 73,902,210–73,902,301 [−]	5′-AGCUUAU-3′
rno-miR-187-3p	MIMAT0000864	18.06	28.21	14.69	1.56	0.52	chr18: 16,390,507–16,390,610 [−]	5′-CGUGUCU-3′
rno-miR-499-5p	MIMAT0003381	50.17	77.94	49.90	1.55	0.64	chr3: 151,138,862–151,138,926 [+]	5′-UAAGACU-3′
rno-miR-151-3p	MIMAT0000614	199.05	307.84	189.36	1.55	0.62	chr7: 114,485,547–114,485,643 [−]	5′-UAGACUG-3′
rno-miR-199a-3p	MIMAT0004738	38.68	59.63	33.12	1.54	0.56	chr13: 80,125,487–80,125,596 [+]	5′-CAGUAGU-3′
rno-miR-31a-5p	MIMAT0000810	9.31	5.96	10.80	0.64	1.81	chr5: 107,206,515–107,206,620 [+]	5′-GGCAAGA-3′
rno-miR-138-5p	MIMAT0000844	201.37	117.04	182.95	0.58	1.56	chr19: 11,149,748–11,149,829 [−]	5′-GCUGGUG-3′
rno-miR-380-5p	MIMAT0005308	34.22	18.97	30.84	0.55	1.63	chr6: 133,860,490–133,860,566 [+]	5′-UGGUUGA-3′
rno-miR-34c-5p	MIMAT0000814	226.02	119.97	200.35	0.53	1.67	chr8: 55,492,024–55,492,100 [−]	5′-GGCAGUG-3′
rno-miR-322-5p	MIMAT0001619	26.03	12.60	19.87	0.48	1.58	chrX: 158,148,161–158,148,255 [+]	5′-AGCAGCA-3′
rno-miR-365-3p	MIMAT0001549	19.22	8.93	15.03	0.46	1.68	chr10: 67,079,796–67,079,881 [+]	5′-AAUGCCC-3′
rno-miR-494-3p	MIMAT0003193	5.95	2.53	4.32	0.42	1.71	chr6: 133,864,370–133,864,452 [+]	5′-GAAACAU-3′
rno-miR-346	MIMAT0000596	14.08	5.70	9.28	0.40	1.63	chr16: 11,250,054–11,250,151 [+]	5′-GUCUGCC-3′
rno-miR-206-3p	MIMAT0000879	14.37	5.17	11.71	0.36	2.27	chr9: 26,791,764–26,791,847 [+]	5′-GGAAUGU-3′

Next, we combined the differentially expressed miRNAs prediction targeted genes among three databases (TargetScan, RNAhybrid and Miranda) with the previously published transcriptome data [9] to depict the miRNA/mRNA regulatory network for the treatment of depression by the LBRD standard decoction (Fig. 5B, C). These predicted target mRNAs overlapping differentially expressed genes (DEGs) in the transcriptome were selected for subsequent analysis. Table 3 shows those significantly alternated miRNAs in the LBRD standard decoction group and their predicted-target mRNAs which matched the DEGs in the transcriptome data. For instance, the rno-miR-495 which was down-regulated in the LBRD standard decoction treated group with comparison to the model group, predicted the up-regulated *GAT-3*, *Calml4*, *Tnfaip6*, *Arc*, *Gad1*, and *BDNF* as the target mRNAs. The altered mRNAs and their correspondent miRNAs are shown in Additional file 1: Table S5. Among those predicted altered mRNAs, *Calml4* corresponded with six changed miRNAs (rno-miR-144-3p, rno-miR-151-3p, rno-miR-204-5p, rno-miR-495, rno-miR-499-5p, and rno-miR-879-5p) and *Acvr1c* corresponded with rno-miR-206-3p, rno-miR-31a-5p, rno-miR-346, rno-miR-34c-5p, rno-miR-380-5p, and rno-miR-494-3p. As shown in Fig. 5D, the overlapped mRNAs were attributed to the neurotrophic signal pathway, mitogen-activated protein kinase (MAPK) signal pathway, PI3K-Akt, cyclic adenosine monophosphate (cAMP), alanine, aspartate and glutamate metabolism, GABAergic synapse, and circadian rhythm etc. by Kyoto Encyclopedia of Genes and Genomes (KEGG) analysis (Additional file 1: Table S6).

**Table 3:** Table 3: The changed mRNAs are regulated by miRNAs in CUMS-induced rat depression model under LBRD standard decoction treatments

miRNAs	The predicted target mRNAs that match DEGs in transcriptome^a^
rno-miR-879-5p ↓	Calml4 ↑ Arc ↑ Gad1 ↑
rno-miR-7a-5p ↓	Slc39a12 ↑ Ntrk2 ↑ GAT-3 ↑
rno-miR-499-5p ↓	Calml4 ↑
rno-miR-495 ↓	GAT-3 ↑ Calml4 ↑ Tnfaip6 ↑ Arc ↑ Gad1↑ Bdnf ↑
rno-miR-204-5p ↓	Calml4 ↑ Ntrk2 ↑ VGAT ↑
rno-miR-199a-3p ↓	Foxs1 ↑ Ntrk2 ↑
rno-miR-151-3p ↓	Calml4 ↑ Ntrk2 ↑
rno-miR-144-3p ↓	Calml4 ↑ Ntrk2 ↑ VGAT ↑ Gad1↑
rno-miR-24-3p ↓	Nr1d1 ↑
rno-miR-25-3p ↓	Btg2 ↑ GAT-3 ↑ VGAT ↑
rno-miR-21-5p ↓	Btg2 ↑ Ntf3 ↑
rno-miR-494-3p ↑	Acvr1c ↓ Syt6 ↓
rno-miR-380-5p ↑	Acvr1c ↓
rno-miR-365-3p ↑	Syt6 ↓
rno-miR-34c-5p ↑	Flot2 ↓ Acvr1c ↓ Syt6 ↓ Il18bp ↓
rno-miR-346 ↑	Dbp ↓ Acvr1c ↓
rno-miR-31a-5p ↑	Flot2 ↓ Acvr1c ↓ Syt6 ↓
rno-miR-206-3p ↑	Dbp ↓ Acvr1c ↓

^a^The target predicted by TargetScan, Rnahybrid and Miranda, and then overlapped to DEGs in transcriptome. ↓, miRNA significantly down-regulated in LBRD standard decoction group *vs* CUMS group, and CUMS group *vs* control group; ↑, up-regulation

In order to confirm the results of miRNA sequencing analysis, three down-regulated miRNAs (rno-miR-144-3p, rno-miR-495, and rno-miR-879-5p) and two up-regulated (rno-miR-34c-5p, and rno-miR-346), were selected for qRT-PCR. Consistent with high-throughput sequencing, these miRNAs were significantly altered in qRT-PCR from the LBRD standard decoction treatment group than those in CUMS-induced depression-like rats (Fig. 5E-F). These findings validated the miRNA sequencing results. By sequencing and bioinformatics analysis, we depicted the regulatory network of miRNA/mRNA for the LBRD standard decoction treatment ameliorating depressive symptoms.

### Effects of LBRD standard decoction on CORT-induced PC12 cells depression model

**Fig. 6:**
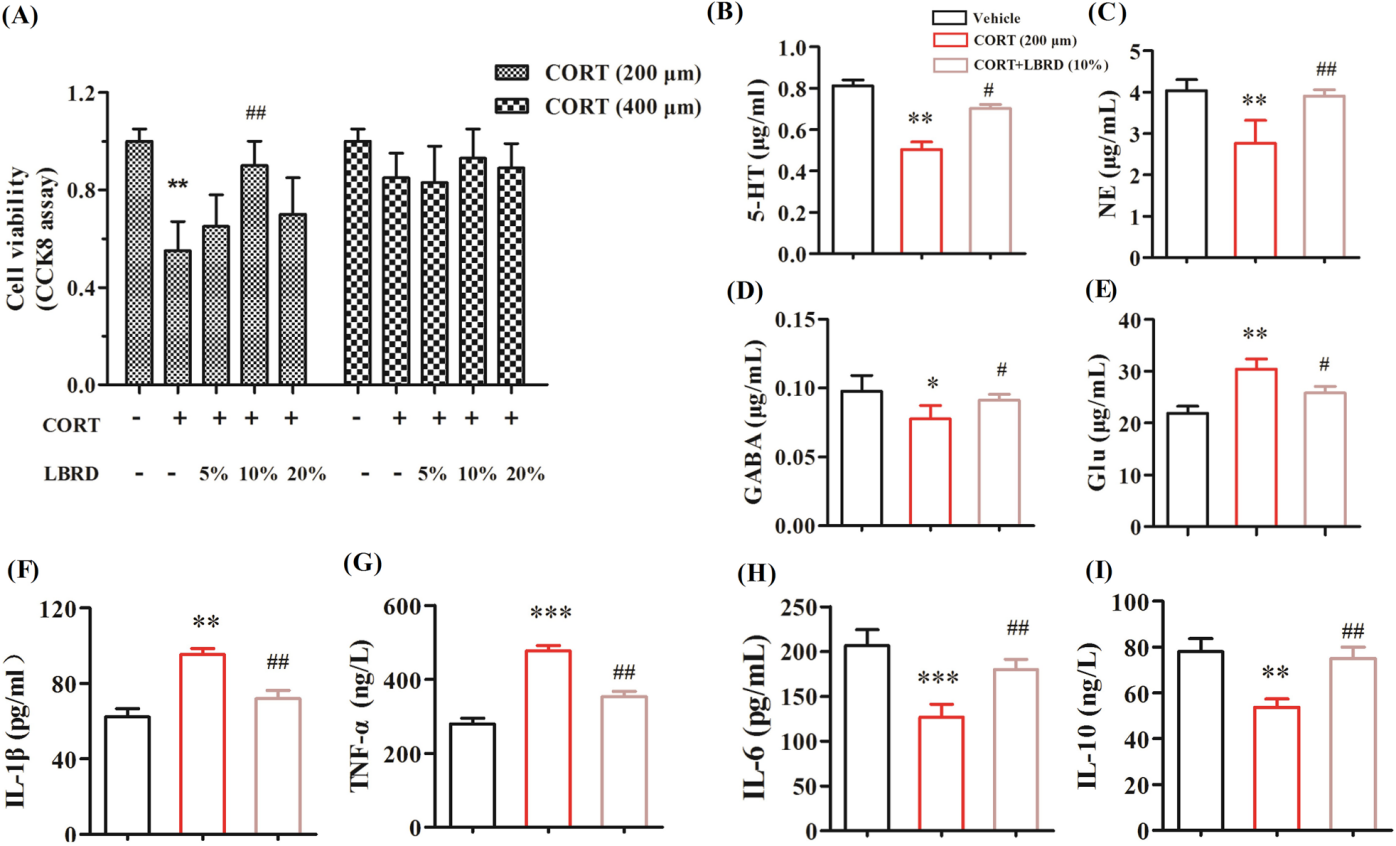
LBRD standard decoction protects PC12 cells against CORT-induced injury. **A** PC12 cells were exposed to CORT (200 and 400 μM) CORT for 24 h and then with LBRD standard decoction-containing serum (5%, 10%, and 20%). Cell viability was determined by CCK-8 assay (n = 6). Quantification of monoamine neurotransmitters 5-HT (**B**), NE (**C**), GABA (**D**), Glu (**E**) and inflammatory cytokine IL-1β (**F**), TNF-α (**G**), IL-6 (**H**) and IL-10 (**I**), in cell culture supernatant (n = 6) harvested from 200 μM CORT-induced PC12 cell depression model intervened with LBRD standard decoction. Data are presented as mean ± SEM and differences between two groups were compared by Student’s *t*-test. ^*^P < 0.05, ^**^P < 0.01, ^***^P < 0.001, compared to the vehicle; ^#^*P* < 0.01, ^##^*P* < 0.01, ^###^*P* < 0.001, compared to the CORT group

The neurotoxicity of PC12 cells can be induced by high concentrations of CORT, which has been extensively used as an in vitro model to investigate the impairment of neurons and depression-like syndromes. Following CORT stimulation (200 and 400 μM) for 24 h, PC12 cells were treated with increased concentrations of LBRD standard decoction-containing serum (5%, 10%, and 20%) for 24 h. Treatment with 200 μM of CORT for 24 h resulted in a decrease of cell viability to approximately 50%. No significant changes were observed in the viability of cells treated with 5% and 20% LBRD standard decoction-containing serum, while treatment with 10% resulted in an obvious effect on cell viability (Fig. 6A, P < 0.05). Thus, in subsequent studies, 200 μM CORT-induced PC12 cells depression model was intervened with 10% concentration of the LBRD standard decoction medicated serum.

Compared to vehicle cells, the levels of monoamine neurotransmitters (5-HT, NE, and GABA) and inflammatory cytokines (IL-1β, IL-6, and TNF-α) were significantly decreased, but Glu and IL-10 were increased in the CORT-induced cell depression-like model. These alterations were genetically consistent with those of CUMS-induced depressed rats and could be resorted under the LBRD standard decoction-containing serum treatment (Fig. 6B–I, both *P* < 0.01). These evidence suggest that the LBRD standard decoction could protect PC12 cells against CORT-induced injury.

### Identification of the miRNA/mRNA regulatory network for LBRD standard decoction protection in CORT-induced PC12 cell depression model

**Fig. 7:**
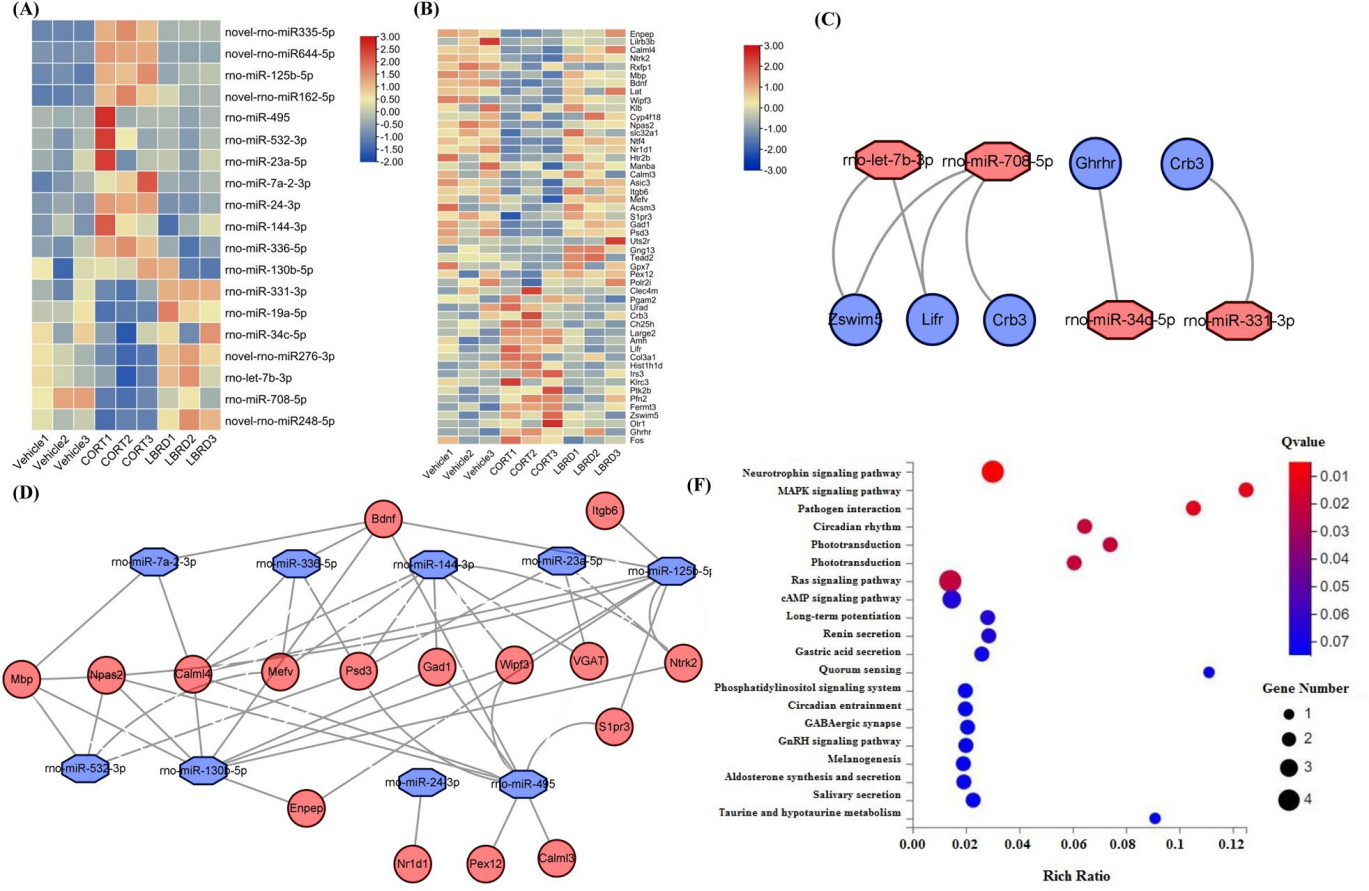
The miRNA/mRNA regulatory network of LBRD standard decoction-containing serum treatment for CORT-induced PC12 cell depression model. **A** Heat map of differentially expressed miRNAs in each PC12 group (n = 3). **B** Heat map of differentially expressed mRNAs in each PC12 group (n = 3). Java Tree View were applied to generate a list of genes based on thermograph to visualize the gene expression profiles in each group. The color changes from red to blue corresponding to expression level changes from high to low, respectively. **C**, **D** Interactive network of miRNA/mRNA for LBRD standard decoction treatment for CORT-induced PC12 cell depression model. Blue represents down-regulated miRNAs and up-regulated mRNAs from the LBRD standard decoction-containing serum in comparison to the CORT group, as well as the CORT-treated cells compared to vehicle cells. Red represents up-regulated miRNAs and down-regulated mRNAs. **E** KEGG pathway of those predicted target mRNAs overlapped with DEGs from transcriptome data about LBRD standard decoction-containing serum treatment for CORT-induced PC12 cell depression model

Similarly, we conducted a detailed transcriptome and bioinformatics analysis of miRNA and mRNA expression to recognize the key genes, pathways and miRNA/mRNA regulatory networks, in the LBRD standard decoction-containing serum against CORT-induced PC12 injury (n = 3). Using an FC of ≥ 1.5, FDR of ≤ 0.05 as well comparing CORT/vehicle and LBRD/CORT with opposite alterations, as thresholds, fourteen known miRNAs and five novel miRNAs were found to be significantly and differentially expressed in each group (Table 4). Among them, twelve different kinds of miRNAs (rno-miR-125b-5p, rno-miR-495, rno-miR-144-3p, rno-miR-24-3p, novel-rno-miR-335-5p, etc.) were down-regulated and seven miRNAs (rno-miR-331-3p, rno-miR-34c-5p, rno-let-7b-3p, and novel-rno-miR-248-5p, etc.) were up-regulated in PC12 cells treated with LBRD standard decoction-medicated serum compared to those from the CORT-induced PC12 cells depression model (Fig. 7A).

**Table 4:** The characteristics of differentially expressed miRNAs in each group of PC12 cells

miRNA	Accession No	Vehicle Means	CORT Means	LBRD Means	Fold change (CORT/Vehicle)	Fold change (LBRD /CORT)	Chromosomal location	Seed sequence
rno-miR-125b-5p	MIMAT0000830	18.61	73.85	36.00	3.97	0.49	chr8: 45,798,260–45,798,346 [+]	5′-CCCUGAG-3′
rno-miR-495	MIMAT0005320	260.55	573.01	252.79	2.20	0.44	chr6: 133,868,288–133,868,367 [+]	5′-AACAAAC-3′
rno-miR-532-3p	MIMAT0005323	14.43	30.51	15.22	2.11	0.50	chrX: 16,109,870–16,109,948 [+]	5′-CUCCCAC-3′
rno-miR-23a-5p	MIMAT0004712	32.47	55.43	36.11	1.71	0.65	chr19: 25,318,582–25,318,656 [+]	5′-GGGUUCC-3′
rno-miR-7a-2-3p	MIMAT0017091	5.53	9.24	6.09	1.67	0.66	chr1: 140,576,396–140,576,490 [+]	5′-AACAAGU-3′
rno-miR-24-3p	MIMAT0000794	549.22	830.35	540.50	1.51	0.65	chr17: 823,968–824,035 [+]	5′-GGCUCAG-3′
rno-miR-144-3p	MIMAT0000850	45.24	74.85	46.67	1.65	0.62	chr10: 65,291,365–65,291,447 [−]	5′-ACAGUAU-3′
rno-miR-336-5p	MIMAT0000576	55.82	87.03	50.96	1.56	0.59	chr10: 35,349,756–35,349,851 [+]	5′-CACCCUU-3′
rno-miR-130b-5p	MIMAT0017122	1.48	2.25	1.36	1.52	0.60	chr11: 88,129,773–88,129,854 [+]	5′-CUCUUUC-3′
novel-rno-miR-335-5p	–	2.47	37.27	13.99	15.08	0.38	–	–
novel-rno-miR-644-5p	–	3.48	25.47	6.32	7.33	0.25	–	–
novel-rno-miR-162-5p	–	64.07	171.22	106.81	2.67	0.62	–	–
rno-miR-331-3p	MIMAT0000570	2.93	1.86	5.85	0.63	3.14	chr7: 34,881,095–34,881,190 [−]	5′-CCCCUGG-3′
rno-miR-19a-5p	MIMAT0017098	1.97	1.23	2.43	0.63	1.98	chr15: 100,180,162–100,180,243 [+]	5′-CGUUUUG-3′
rno-miR-34c-5p	MIMAT0000814	45.78	27.97	47.73	0.61	1.71	chr8: 55,492,024–55,492,100 [−]	5′-GGCAGUG-3′
rno-let-7b-3p	MIMAT0004705	5.45	2.73	6.93	0.50	2.54	chr7: 126,590,627–126,590,711 [+]	5′-UAUACAA-3′
rno-miR-708-5p	MIMAT0005331	10.59	5.16	8.18	0.49	1.59	chr1: 161,221,246–161,221,333 [+]	5′-AGGAGCU-3′
novel-rno-miR-248-5p	–	8.60	3.14	13.22	0.37	4.21	–	–
novel-rno-miR-276-3p	–	12.82	7.63	16.29	0.60	2.13	–	–

For mRNA sequencing analysis, thirty-one mRNAs were significantly up-regulated in the PC12 cells from the LBRD standard decoction-containing serum group, and nineteen mRNAs were down-regulated (Table 5). After LBRD standard decoction-containing serum administration of the CORT-induced cell depression model, there were remarkably increased expressions of mRNAs including *Calml4*, *Ntrk2*, *Bdnf*, *Npas2*, *Mbp*, *Wipf3*, *VGAT*, *Nr1d1*, *Tead2 and Gad1*; whereas compared to the vehicle group, *Fos*, *Hist1h1d*, *Ptk2b, Urad*, *Ch25h*, and *Lifr* mRNA were obviously decreased (Fig. 7B). These DEGs were significantly enriched in the following signaling pathways and processes, such as the cAMP neurotrophic, MAPK, and calcium signaling pathways, circadian rhythm, dopaminergic synapse, and neuroactive ligand-receptor interaction (Additional file 1: Figure S3 and Table S7).

**Table 5:** The characteristics of differentially expressed mRNAs in each group of PC12 cells

Gene symbol	Gene ID	Length (bp)	Chromosomal map	Fold change (Vehicle /CORT)	Fold change (LBRD/CORT)	Description
Enpep	64,017	69,962	2q42	0.25	3.38	Glutamyl aminopeptidase
Lilrb3b	690,955	7471	1q12	0.26	2.00	Leukocyte immunoglobulin-like receptor, subfamily B (with TM and ITIM domains), member 3B
Calml4	691,455	11,808	8q24	0.40	2.53	Calmodulin-like 4
Ntrk2	25,054	311,307	17p14	0.42	2.29	Neurotrophic receptor tyrosine kinase 2
Rxfp1	295,144	121,214	Lgr7	0.44	0.64	Relaxin family peptide receptor 1
Mbp	24,547	110,526	18q12.3	0.45	2.04	Myelin basic protein
Bdnf	24,225	50,579	3q34	0.46	1.76	Brain-derived neurotrophic factor
Lat	81,511	5025	1q36	0.48	2.08	Linker for activation of T cells
Wipf3	259,242	79,854	4q24	0.50	1.75	WAS/WASL interacting protein family, member 3
Klb	289,625	51,738	14p11	0.51	1.84	Klotho beta
Cyp4f18	290,623	41,401	16p14	0.52	2.13	Cytochrome P450, family 4, subfamily f, polypeptide 18
Npas2	316,351	178,961	9q22	0.52	1.51	Neuronal PAS domain protein 2
VGAT	83,612	4469	3q42	0.55	1.62	Solute carrier family 32 member 1
Ntf4	25,730	2831	1q22	0.56	1.79	Neurotrophic 4
Nr1d1	252,917	7214	10q31	0.57	1.57	Nuclear receptor subfamily 1, group D, member 1
Htr2b	29,581	20,847	9q35	0.58	1.61	5-hydroxytryptamine receptor 2B
Manba	310,864	92,556	2q43	0.58	1.62	Mannosidase beta
Calml3	307,100	3239	17q12.2	0.58	1.57	Calmodulin-like 3
Asic3	286,920	4478	4q11	0.59	1.72	Acid sensing ion channel subunit 3
Itgb6	311,061	124,237	3q21	0.59	1.69	Integrin subunit beta 6
Mefv	58,923	10,029	10q12	0.59	2.00	MEFV, pyrin innate immunity regulator
Acsm3	24,763	26,706	1q35	0.60	2.16	acyl-CoA synthetase medium-chain family member 3
S1pr3	306,792	13,358	17p14	0.61	1.58	Sphingosine-1-phosphate receptor 3
Gad1	24,379	40,631	3q22	0.61	1.64	Glutamate decarboxylase 1
Psd3	306,380	570,203	16p14	0.63	1.51	Pleckstrin and Sec7 domain containing 3
Uts2r	57,305	1160	10q32.3	0.64	1.97	Urotensin 2 receptor
Gng13	685,451	1904	10q12	0.65	3.56	G protein subunit gamma 13
Tead2	308,582	16,820	1q22	0.66	1.90	TEA domain transcription factor 2
Gpx7	298,376	7990	5q34	0.67	1.56	Glutathione peroxidase 7
Pex12	116,718	8036	10q26	0.67	2.33	Peroxisomal biogenesis factor 12
Polr2i	292,778	1437	1q21	0.67	1.51	RNA polymerase II subunit I
Clec4m	288,378	8627	12p12	1.53	0.47	C-type lectin domain family 4 member M
Pgam2	24,959	2111	14q21	1.55	0.56	Phosphoglycerate mutase 2
Urad	684,055	9692	12p11	1.67	0.43	Ureidoimidazoline (2-oxo-4-hydroxy-4-carboxy-5-) decarboxylase
Crb3	301,112	5329	9q12	1.69	0.57	Crumbs 3, cell polarity complex component
Ch25h	309,527	1318	1q53	1.75	0.33	Cholesterol 25-hydroxylase
Large2	311,202	12,540	3q24	1.81	0.53	LARGE xylosyl- and glucuronyltransferase 2
Amh	25,378	2408	7q11	1.81	0.62	Anti-Mullerian hormone
Lifr	81,680	68,595	2q16	1.82	0.43	LIF receptor alpha
Col3a1	84,032	35,936	9q22	1.83	0.63	Collagen type III alpha 1 chain
Hist1h1d	201,097	659	17p11	1.87	0.43	Histone cluster 1, H1d
Irs3	84,021	2138	12q12	1.93	0.61	Insulin receptor substrate 3
Klrc3	500,338	7656	4q42	1.93	0.21	Killer cell lectin like receptor C3
Ptk2b	50,646	120,513	15p12	2.06	0.50	Protein tyrosine kinase 2 beta
Pfn2	81,531	5836	2q26	2.08	0.67	Profilin 2
Fermt3	309,186	18,200	1q43	2.16	0.61	Fermitin family member 3
Zswim5	313,524	117,272	5q36	2.23	0.55	Zinc finger, SWIM-type containing 5
Olr1	140,914	22,621	4q42	2.40	0.53	Oxidized low density lipoprotein receptor 1
Ghrhr	25,321	34,688	4q24	3.98	0.68	Growth hormone releasing hormone receptor
Fos	314,322	2866	6q31	4.25	0.19	Fos proto-oncogene, AP-1 transcription factor subunit

Subsequently, we constructed the miRNA/mRNA regulatory network for LBRD standard decoction treatment of the CORT-induced PC12 cell depression model (Fig. 7C, D). The candidate mRNAs should be predicted by differentially expressed miRNAs and also overlapped to DEGs in the PC12 cell transcriptome. Table 6 showes the alternated miRNAs and their predicted-target mRNAs. For example, the downregluated rno-miR-144-3p predicted the up-regulated *Gad1*, *Ntrk2*, *Mefv*, *VGAT*, *Psd3*, *Wipf3*, and *Calml4* as potential targets, whereas the up-regulated rno-miR-708-5p adsorbed on the 3′-untranslated region (UTR) of the down-regulated *Zswim5*, *Crb3*, and *Lifr* mRNAs. The altered mRNAs in the LBRD standard decoction-treated cell depression group and their corresponding miRNAs are listed in Additional file 1: Table S8. Among those predicted differently expressed mRNAs, *Bdnf* was predicted by the rno-miR-495, rno-miR-125b-5p, rno-miR-336-5p, rno-miR-7a-2-3p, and rno-miR-130b-5p.

**Table 6:** The differently expressed mRNAs in CORT-induced cell depression model treated with LBRD standard decoction were regulated by miRNAs

miRNAs	The predicted target mRNAs that match DEGs in transcriptome^a^
rno-miR-7a-2-3p ↓	Mbp ↑ Bdnf ↑ Calml4 ↑
rno-miR-532-3p ↓	Mbp ↑ Mefv ↑ Psd3 ↑ Npas2↑
rno-miR-495 ↓	Gad1↑ Bdnf ↑ Pex12 ↑ Wipf3 ↑ Calml3 ↑ S1pr3 ↑ Psd3 ↑ Calml4 ↑ Npas2 ↑
rno-miR-336-5p ↓	Bdnf ↑ Mefv ↑ Psd3 ↑ Calml4↑
rno-miR-24-3p ↓	Nr1d1↑
rno-miR-23a-5p ↓	Ntrk2 ↑ VGAT ↑ Psd3 ↑
rno-miR-144-3p ↓	Gad1↑ Ntrk2 ↑ Mefv ↑ VGAT ↑ Wipf3 ↑ Psd3 ↑ Calml4↑
rno-miR-130b-5p ↓	Mbp ↑ Gad1↑ Bdnf ↑ Ntrk2 ↑ Enpep ↑ Wipf3 ↑ Calml4 ↑ Npas2↑
rno-miR-125b-5p ↓	Mbp ↑ Bdnf ↑ Ntrk2 ↑ Enpep ↑ Wipf3 ↑ Itgb6 ↑ S1pr3 ↑ Calml4 ↑
rno-let-7b-3p ↑	Zswim5 ↓ Lifr↓
rno-miR-708-5p ↑	Zswim5 ↓ Crb3 ↓ Lifr↓
rno-miR-34c-5p ↑	Ghrhr↓
rno-miR-331-3p ↑	Crb3↓

^a^The target mRNAs predicted by TargetScan, Rnahybrid and Miranda, and then overlapped to DEGs in transcriptome. ↓, miRNA significantly down-regulated in LBRD standard decoction-containing serum group *vs* CORT group, and CORT group *vs* vehicle group; **↑**, up-regulation

Finally, KEGG pathways analysis was performed for these predicted target mRNAs which also overlapped to DEGs in the cells transcriptome (Additional file 1: Table S9). The neurotrophic, RAS, and cAMP signaling pathways, circadian rhythm, long-term potentiation, and GABAergic synapse were implicated in the LBRD standard decoction-containing serum protection of the CORT-induced PC12 cell depression model (Fig. 7E).

### Verification of the role of miRNA/mRNA regulatory network-mediated GABA and BDNF expression in LBRD standard decoction action on depression

**Fig. 8:**
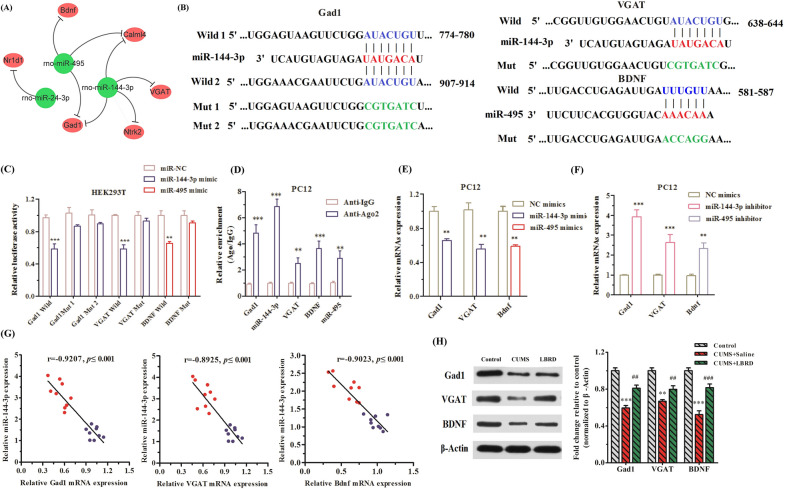
Antidepressant effect of LBRD standard decoction on the mRNA/miRNA regulatory network of GABA and BDNF expression. **A** The interactive networks of four overlapped miRNAs and six mRNAs in the LBRD standard decoction treated group from RNA sequencing data of CUMS-induced animal and CORT-induced cell depression model. Green symbols denote miRNAs that were downregluated with LBRD standard decoction treatment, and red symbols denote up-regulated mRNAs. Their negative regulation of mRNAs by miRNAs is represented by a grey line. **B** The specific binding regions between miR-144-3p and Gad1/VGAT, and miR-495 and *Bdnf* were obtained from the online software database. **C**, **D** The relationship between miR-144-3p and *Gad1/VGAT*, and miR-495 and Bdnf were confirmed by dual-luciferase reporter and RIP assays (n = 3). Normal immunoglobulin was used as a negative control. **E**, **F** qRT-PCR analysis of *Gad1, VGAT*, and *Bdnf* in PC12 cells respectively transfected with miR-144-3p and miR-495 mimics or inhibitor. **G** Association of miR-144-3p, miR-495 and its corresponding target genes were assessed by Pearson’s correlation coefficients with qRT-PCR results (n = 9 pre/group). Blue dots indicate congregation of the LBRD standard decoction-treated group and red dots indicate congregation of the depression group. **H** The expression level of Gad1, VGAT, and BDNF proteins were measured in mPFCs tissues obtained from LBRD standard decoction-treated group. ^**^P < 0.01, ^***^P < 0.001, compared to control group; ^##^*P* < 0.01, ^###^*P* < 0.001, compared to CUMS + Saline group. CUMS + Saline: CUMS + Saline group, CUMS + LBRD: CUMS + Lily Bulb and Rehmannia Decoction group

When, comparing the RNA sequencing date from CUMS-induced animals and CORT-induced cell depression model, there were four overlapped miRNAs (rno-miR-144-3p, rno-miR-495, rno-miR-34c-5p, and rno-miR-24-3p) and six mRNAs (*Calml4, Ntrk2, VGAT, Gad1, Nr1d1,* and *Bdnf*), in the LBRD standard decoction-treated group. Among them, rno-miR-144-3p predicted *Calml4*, *Ntrk2*, *VGAT*, *and Gad1* as potential targets; rno-miR-495 targeted *Calml4*, *Gad1*, and *Bdnf*, and *Nr1d1* mRNA 3′-UTR was predicted as the rno-miR-24-3p binding site (Fig. 8A). Regrettably, there was no predictable correlation between rno-miR-34c-5p and its mRNAs.

To verify the mRNA/miRNA regulatory network for GABA and BDNF expression, we selected rno-miR-144-3p and rno-miR-495 to examine whether they targeted *Gad1*, *VGAT*, and *Bdnf* by dual luciferase reporter and RIP assay. The data showed that over-expression of miR-144-3p could reduce the luciferase activity of the wild-type Gad1 and VGAT reporters, but it did not affect the luciferase activity of the corresponding mutation vector in HEK293T cells. In addition, miR-495 mimics and BDNF 3′-UTR wild type co-transfection caused the decreased luciferase activity in HEK293T cells relative to NC mimics, while miR-495 mimics and BDNF 3′-UTR mutant co-transfection did not alter the luciferase activity (Fig. 8B, C).

In addition, the RIP assay exhibited that miR-144-3p and *Gad1*, and *VGAT* could notably be loaded in the AGO2 RIP but not IgG RIP in PC12 cells. Similarly, miR-495 and *Bdnf* were both significantly enriched in Ago2-containing beads compared with the IgG-containing beads (Fig. 8D). Next, PC12 cells were separately transfected with miR-144-3p, miR-495 or NC-mimics, and qRT-PCR analysis revealed that over-expression of miR-144-3p or miR-495 could suppressed *Gad1*, *VGAT*, and *Bdnf* expression, respectively (Fig. 8E), while the inhibition of the corresponding miRNAs could increase the expression of their target genes (Fig. 8F).

Finally, we performed Pearson's correlation test of miRNA/mRNA regulatory GABA- associated genes and *Bdnf* expression. The linear regression analysis showed that miR-144-3p and miR-495 were negatively correlated with the expression of *Gad1*, *VGAT* and *Bdnf* mRNAs, respectively (Fig. 8G). Similarly, the protein expressions of Gad1, VGAT, and BDNF in the mFPC tissues were markedly increased (Fig. 8H) in the saline-treated group compared to the LBRD standard decoction-administrated group. Taken together, the LBRD standard decoction exerted an antidepressant effect by regulating the miRNA/mRNA network-mediated GABA and BDNF expression.

## Discussion

Currently, an outbreak of coronavirus disease 2019 (COVID-19) infected by severe acute respiratory syndrome coronavirus 2 (SARS-CoV-2), is still spreading and has led to an unprecedented health crisis worldwide [24]. In response to this pandemic, the Chinese government proposes that integrated TCM and WM (also known as integrated medicine) can be applied to treat pneumonia caused by SARS-CoV2 [25]. In fact, prevention, treatment, and rehabilitation of the present COVID-19 situation in China could not be separated from the extensive participation of TCM, which has proven effective for SARS-CoV-2 by clinical and laboratory studies [7, 26–28]. TCM therapies, such as Chinese herbal formulas, acupuncture, massage, etc., have been widely applied to treat epidemics for over a thousand years under the guideline of TCM theories such as yin and yang, five elements theory, and meridians and collaterals theories [29].

The outstanding curative effect of TCM formula or prescription for COVID-19 has attracted the global research community's attention towards TCM. Scientists frequently find new therapeutic agents for various diseases in TCM formulas or herbs. TCM formulas, usually composed of different Chinese herbs based on the Chinese materia medical theory (four natures, five flavors and meridian tropism), could comprehensively improve organ function through multi-components acting on multi-targets at multi-pathways [30]. The TCM formula is currently not only used prevalently in Asian countries but has also gained a global market. In Western countries, it has been gradually recognized as a popular form of complementary and alternative medicine with the belief that the TCM formula incurs fewer side effects, as it is generally composed of natural products without artificial additives.

Due to the unique philosophy, diagnosis, prescription principles and processing methods, TCM formulas are quite different from WM drugs. The applicability of ancient TCM formula on modern disease, the applicable quality standards, and the synergistic mechanism behind its efficacy, as well as its development in a modernized industry manner, have faced great challenges [31]. In ancient China, depressive syndrome is classified as an "emotional diseases" and is closely related to the manifestations of "lily disease", "epilepsy", "insomnia", "hysteria", "sensation of qi rushing", and so on. From the TCM perspective, we have verified that the symptoms of "lily disease" are generally similar to the clinical manifestations of depression, and the LBRD is the specialized formula for it [5]. As a TCM classical formula for resolving depression and tranquilizing the mind, LBRD exhibits pharmacological effects on nourishing the lung, arresting sweating, calming the heart, and promoting fluid production [32]. In addition, emerging evidence has indicated that LBRD can significantly ameliorate the states of depression and anxiety [33, 34]. All the findings involve the use of ancient TCM formulas to treat modern disease, and provide a reference for the LBRD clinical application and future development.

In the long history of China, the unit for weights and measures has changed greatly, and therefore it is inevitable that the dosage of herbs or formulas used in ancient and modern times is quite different. Furthermore, TCM researches have varied views about the original dosage and genuine production area of TCM herbs. These controversial issues have affected the application of LBRD in clinic. Through literature research on materia medica, dosage, processing, and production area, we have revealed the preparation and development of LBRD, found out the genuine lily bulb, raw Rehmannia, and spring water, and established the procedure for LBRD standard decoction preparation [9]. We propose to establish a traceability database, which will be beneficial for LBRD development and modernization, and will makes it available for the global market.

Currently, the active components in the LBRD standard decoction and the corresponding chemical spectrums have not been determined. In the current study, we used UHPLC-Q-TOF/MS to identify the chemical profiling and bioactive compounds of the LBRD standard decoction for the first time. In total, 32 prototype compounds in the LBRD standard decoction were detected and the major ingredients were polysaccharides, phenolic acid glycerides, glycosides, iridoid glycosides, etc. (Fig. 1). Among them, twenty five representative constituents (verbascoside, kojibiose, qsmundalactone, triethyl phosphate, myristic acid, etc.) were all detected in the lily bulb decoction, Rehmannia root juice, and the LBRD standard decoction. The quantitative analysis of verbascoside using HPLC suggested the contents were in line with the standard qualities of lily bulb and Rehmannia root in the *Pharmacopoeia of People's Republic of China* (2015 Edition). In terms of the transmission of ingredients, the changing trend of verbascoside was elevated from fresh lily bulb decoction and raw Rehmannia juice to the LBRD standard decoction.

Verbascoside, a phenypropanoid glycoside, has been demonstrated to exert antidepressant and anxiolytic effects through modulation of cAMP, calcium homeostasis (calcium channels), and energy metabolism [35–37]. Our behavior test results indicted verbascoside at 60 mg/kg displayed anxiolytic and antidepressant activity (Additional file 1: Figure S4). Additionally, the treatment effect exerted antidepressant activity similar to fluoxetine and the LBRD standard decoction. Thus, verbascoside could not only serve as the quality marker for the LBRD standard decoction, but also as an antidepressant bioactive ingredient. In the future, it is necessary to comprehensively identify the potential components and signaling pathways which could be applied for treating depression, and to understand the relationship between the LBRD standard decoction components and the efficacy.

Although LBRD has been commonly used for the treatment of mental diseases for thousands of years, there is still a lack of scientific evidence to support its efficacy in many cases. Various factors (origin, geoherbalism, and environmental factors) will affect the chemical composition and content of active integrands in LBRD, and even lead to fluctuating therapeutic effects. So, the LBRD standard decoction in the current study was produced according to the standard preparation procedures and product quality was evaluated based on the effects of treating stress-induced depression. According to the behavior test results, we could concluded that the LBRD standard decoction has an anxiolytic and antidepressant effect (Fig. 2). Interestingly, the therapeutic efficacy of the LBRD standard decoction was close to that of fluoxetine (Prozac), which is a commonly used clinical treatment for depression treatment in clinic. However, meta-analyses suggest that fluoxetine is effective for only one-half to one-third of patients suffering from depression [38, 39]. In addition to time lag and long-term medication, it brings about numerous undesirable side effects (including acute nausea and headaches, as well as chronic sexual dysfunction and diminished rapid eye movement sleep), and also increases the economic burden of patients [40–42]. In contrast, the LBRD standard decoction possesses various chemical components, multi-targets, as well as multiple pharmacological effects, which perform synergistically while avoiding the side effects caused by single link action [32, 43–46]. Furthermore, each single herb of the LBRD standard decoction is beneficial for central nervous system disorders and widely used in an Asian-medicated diet [47–49]. Therefore, the LBRD standard decoction may serve as a promising antidepressant therapeutic agent with strong efficacy and high safety.

In fact, the multi-component and multi-target mechanism of the TCM formula is fundamentally different from the mono-substance and mono-target model of WM drugs. Therefore, using the protocols from WM to evaluate the efficacy of the LBRD standard decoction would be inadequate or inefficient. In a previously published report, we summarized the clinical symptoms, etiology, pathogenesis, and TCM formula of “lily disease”. We stated that the underlying mechanism for depression of internal heat was due to yin deficiency, and discussed the relationship between “lily disease” and depression of internal heat [8]. The cause of lily disease is the damage of viscera by external or internal injury, which would lead to yin deficiency of viscera, and the disorder of qi and blood. As a result, the heart would lose nourishment, and hence, there would be symptoms of mental disorder. Finally, yin deficiency of viscera will lead to deficient heat generation inside the body, manifesting as bitterness in the mouth, dark urine, and other symptoms. Therefore, we could infer that internal heat depression due to yin deficiency is a major symptom of “lily disease” based on the etiology and pathogenesis. Combining CUMS and warm-heat TCM herbs we established a rat model of internal heat depression due to yin deficiency, whose manifestation resembled that of “lily disease”. Our results showed that the LBRD standard decoction had significant effects on this rat model (Additional file 1: Figure S5). Therefore, we tentatively concluded that based on the holistic concept of TCM and the principles of syndrome differentiation, we established a depression model of yin deficiency and internal heat syndrome. This study would provide a research method suitable for the investigation into the complex nature of TCM formula.

As of now, multiple studies allowed the formulation of several theories attempting to elucidate the pathogenesis of depression, including the monoamine [50], cytokine [51], HPA hyperactivity [52], neuroinflammation and neuroplasticity [53], GABA-glutamate-mediated depression [54], and circadian hypotheses [55]. These theoretical studies show that depression is a complex mental disorder involving a variety of neurotransmitters, brain regions, circuits and various biochemical substances or/and systems. In the current study, we showed that the LBRD standard decoction administration inhibited the decrease in the levels of monoamine neurotransmitter, inhibitory neurotransmitter (GABA), and pro-inflammatory cytokines, while decreasing the levels of the excitatory neurotransmitter (Glu) and anti-inflammatory cytokines (IL-10) release (Fig. 3). Since the hypothalamic CRH and pituitary ACTH or TSH in the central nervous system were also downregulated by the LBRD standard decoction, it seems that the LBRD standard decoction controls the secretion of CORT and T4 in the plasma. CORT, the final effectors of the HPA-axis, is a principal glucocorticoid secreted in response to stress, and it could decrease 5-HT release and lead to neurodegeneration. Our data provided a strong evidence that the LBRD standard decoction ameliorates depressive and anxiety symptoms by abolishing inhibitory/excitatory neurotransmitter deficits, recovering the balance of pro-inflammatory/anti-inflammatory cytokines, and reducing HPA-axis hormone levels.

mPFC, a hub region in the integration and consolidation of emotional responses to chronic stress, is highly impacted in depressive patients and animal models of chronic stress [4, 56]. This complex process appears to involve a series of morphological and anatomical alterations combined with changes in the strength of mPFC inputs from several brain structures [57]. Accumulated evidence from mPFC morphological studies displayed reduced gray matter along with decreased number and length of dendrites in pyramidal neurons, and reduction of density and size in GABA interneurons, were linked to the severity of illness and duration of treatment [58–60]. In a manner similar to a previous study, chronic stress decreased the number of prefrontal cortex neuronal cells and increased the injured myelinated nerve fibers [61, 62]. Noticeably, LBRD standard decoction intervention revised the neuron pathological alterations and myelinated nerve fibers deficits in the mPFC tissues. The LBRD standard decoction imparted a protective effect against the reduction of neuron expression induced by chronic stress. Numerous evidences demonstrate that a decrease in the number of neurons in the prefrontal region reflects the pathology of depression, and it is encouraging to note that treatment with the LBRD standard decoction reversed ablation of neurons [63–65].

Recently, the application of epigenetics, is the frontier research method of mechanisms in neurological system diseases [66–69]. Epigenetic mechanisms, especially miRNA-mediated post-transcriptional mRNA expression regulation, contribute to the occurrence, progression, recurrence, and treatment of depression [70]. Given the fact that the overall and dynamic view of epigenetics shares many similarities with the holistic concept and treatment based on syndrome differentiation in TCM, we tried to decode the epigenetic mechanisms behind the efficacy of the LBRD standard decoction antidepressant through high throughput RNA-sequencing and a series of bioinformatics analyses. We formulated strict screening criteria to ensure that the candidate miRNA or mRNA not only plays an essential role in the development and maintenance of depression, but also serves as therapeutic targets for the LBRD standard decoction. There were eleven down-regulated miRNAs corresponding to thirteen up-regulated mRNAs in mPFC tissues found in the depression rat treated with LBRD standard decoction, while seven up-regulated miRNAs corresponded to five down-regulated mRNAs (Fig. 5, Table 3, Additional file 1: Table S5). These altered miRNAs (rno-miR-879-5p, rno-miR-7a-5p, rno-miR-499-5p, rno-miR-495, rno-miR-204-5p, rno-miR-199a-3p, rno-miR-151-3p, rno-miR-144-3p, rno-miR-24-3p, rno-miR-25-3p, and rno-miR-21-5p) targeting differentially expressed mRNAs (*Btg2*, *Slc39a12*, *Calml4*, *Foxs1*, *Nr1d1*, *Ntrk2*, *Tnfaip6*, *Arc*, *Ntf3*, *GAT-3*, *VGAT*, *Gad1*, and *Bdnf*) were attributed to the neurotrophic, MAPK, PI3K-Akt, and cAMP signaling pathways, alanine, aspartate and glutamate metabolism, GABAergic synapse, and circadian rhythm. These signaling pathways were also involved in the anti-depression and anti-anxiety efficacy of current alternative therapy including TCM formula, Chinese herbs, extract, acupuncture, massage, exercise, and in experimental manipulations with fast-acting antidepressant-like (ketamine) activity [6, 71–76].

Currently, the in vivo depression models include zebrafish, rodents, and nonhuman primates, and among them, the rodent animals are the most widely used in depression studies among them. However, these animal models do not always satisfy the evaluation criteria of depressive phenotype and some models cost too much time and money. Therefore, many researchers have focused their attention on an in vitro model of depression, which has the exact mechanism, as well as speed and economic properties in favor of large-scale drug screening. The cell used for depression studies and antidepressant screening include primary cultured cells, PC12 cells, glioma C6 cells, SH-SY5Y neuroblastoma cells, and others. Previous studies have suggested that high concentrations of CORT can induce cellular injury in PC12 cells [77, 78], which simulate the pathogenesis of depression in vitro, and this can be reversed with antidepressants. Consistent with these findings, 200 μM of CORT treatment for 24 h led to a 50% decrease in the cell viability compared with the control in our study. By contrast, LBRD standard decoction-containing serum (10%) obviously increased the cell viability. Therefore, inhibiting the apoptosis of PC12 cells may be an effective measure to prevent the progression of neurotoxicity. In addition, the altered level of neurotransmitters and inflammatory cytokines in CORT-included PC12 cells were also consist with that in the rats depression model, while the LBRD standard decoction acted it. These results strongly confirm PC12 damaged by high concentrations CORT as the suitable in vitro depression model and support LBRD standard decoction attenuates CORT-induced PC12 cells injury by inhibition of apoptosis and modulation of homeostasis between inhibitory and excitatory neurotransmitters.

We further explored the possible mechanisms underlying the protective effect of LBRD standard decoction through adopting the same sequencing and screening methods as in vivo animal model research. To our knowledge, this is the first time to reveal the miRNA/mRNA regulatory network of the LBRD standard decoction is revealed to counteract CORT-induced cell depression-like behavior. These perturbed miRNAs (rno-miR-7a-2-3p, rno-miR-532-3p, rno-miR-495, rno-miR-336-5p, rno-miR-24-3p, and rno-miR-144-3p) negatively regulated differently expressed genes (*Mbp, Gad1, Bdnf, Ntrk2, Enpep, Mefv, VGAT, Pex12, Wipf3, Nr1d1*, etc.), which correlated to the neurotrophic, RAS, cAMP signaling pathways, circadian rhythm, long-term potentiation, and GABAergic synapse (Fig. 7). Importantly, both GABAergic synapse and neurotrophic signaling pathways (BDNF-Ntrk2) were involved in the antidepressant and anti-anxiety effects of the LBRD standard decoction in both in vitro and in vivo models. Reduction in BDNF and GABA neurotransmission co-occur in the mPFC tissues and has been repeatedly seen in animal models and human subjects, and this has been associated with anhedonia and anxiety [54, 79–82]. In addition, treatment with antidepressants and anti-anxiety agents, as well as repetitive transcranial magnetic stimulation treatment has been shown to enhance GABA and BDNF expression in depression [83, 84].

We hypothesized that the anti-depressive effects of the LBRD standard decoction may also rely on the prefrontal cortex miRNA-mediated GABA and BDNF expression. At present, the role of miRNAs associated with GABA release and BDNF expression in depressive disorders remain unclear. Evidence from our current luciferase and RIP assay suggested *Gad1* and *VGAT* were directly regulated by binding the seed sequence of miR-144-3p, and miR-495 could be served as a post-transcriptional regulator by binding to the *Bdnf* 3′-UTR. In functional interaction studies, over-expression of miR-144-3p or miR-495 using targeted miRNA mimics were associated with reduced expression of *Gad1*, *VGAT*, and *Bdnf* mRNAs. Not surprisingly, the LBRD standard decoction corrected the increased miR-144-3p and miR-495, whereas inhibited *Gad1*, *VGAT*, and *Bdnf* mRNAs and protein expression in the chronic stress-mediated depression and anxiety model. miR-144-3p targets a number of genes including *Pten*, *Spred1*, *EGFR*, *Nrf2*, *AQP*1, *NGF*, *Brg1* and *Notch*, which are implicated in the stress response, anxiety and mood stabilizer treatment [85–90]. Recent studies showed miR-495 as a novel regulator of multiple addiction-related genes (*Bdnf*, *Camk2a*, and *Arc*) within the nucleus accumbens that have a role in modulating motivation for cocaine [91]. There is a close interaction between BDNF and GABAergic transmission. In inhibitory synapses, both increased and decreased BDNF levels contribute to abnormal GABAergic transmission via alteration of GABA release and GAT-related transport of GABA, and abnormal regulation and decreased transcription of GABAAR [92]. Notably, Toshifumi et al. [93] revealed a novel mechanism by which deficient BDNF leads to targeted reduced GABAergic signaling through autophagic dysregulation of p62, potentially underlying reward and cognitive impairments across brain conditions. Thus, the activity-dependent release of BDNF, which enhances both GABA and glutamate synaptic functions, could be a key mediator of the LBRD standard decoction effects and the epigenetic mechanisms may be related to miR-144-3p and miR-495-mediated GABAergic function and BDNF expression.

Finally, we put forward the integration between genetic or epigenetic mechanism and the overall efficacy of the compound. This is an important approach to the future of TCM and modern medicine could mutually promote their development and would also form a new research model of TCM. However, more effort is needed to minimize the safety risks and provide scientific proof and clinical evaluation for the continuous development of ancient TCM formula therapies. Furthermore, the pharmacological mechanism of the LBRD standard decoction antidepressant in depression-related brain areas and neural circuits need to be studied. More importantly, whether changing the GABA-related miRNA network in GABAergic neurons reverses the therapeutic effect of the LBRD standard decoction. Gratifyingly, the government of China has proposed a series of regulations or laws to simplify the registration procedure of TCM classical formulas. Modern biomedical technology combined with the high-end manufacturing industry in China, will accelerate the modernization of TCM formulas, which will be of benefit to build a community with a shared future for human health.

## Conclusion

**Fig. 9:**
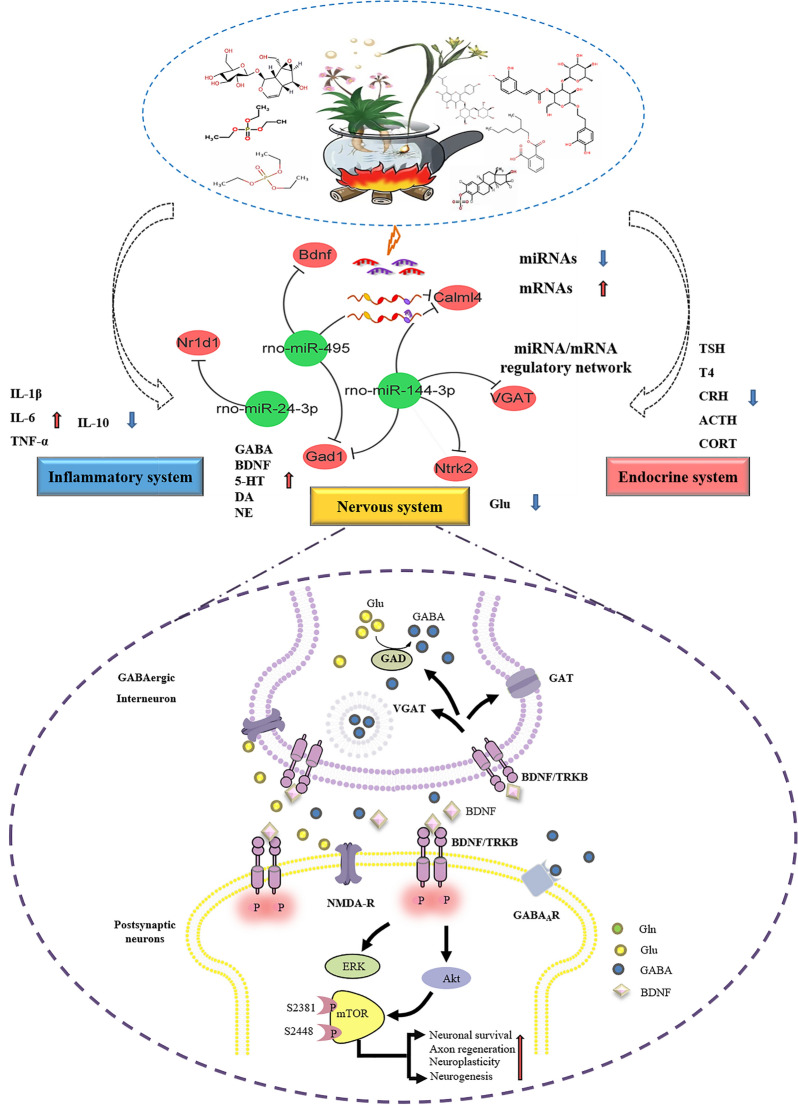
Schematic diagram of integrated analysis of the chemical-material basis and miRNA/mRNA regulatory network for LBRD standard decoction alleviating depression. The nervous, immuno-inflammatory, and endocrine systems were mainly involved in the antidepressant mechanisms of the LBRD standard decoction. Notably, LBRD standard decoction changed a series of miRNAs by which targeted mRNAs correlated to GABAergic synapse, RAS, circadian rhythm and neurotrophic signaling pathways to achieve a homeostatic state of multi-system functions in depression

Collectively, the nervous, immuno-inflammatory, and endocrine systems were mainly related to the antidepressant mechanisms of the LBRD standard decoction. The multi-components of the LBRD standard decoction altered a series of miRNAs to mediate GABAergic synapse, circadian rhythm, RAS and neurotrophic signaling pathways, thereby abolishing inhibitory/excitatory neurotransmitter deficits, as well as restoring the pro-inflammatory/anti-inflammatory cytokine levels and controlling the HPA-axis hormone secretion to achieve balance of multi-system functions in depressed patients (Fig. 9). In the current study, the material basis, bioactive ingredients, and pharmacological mechanisms of the LBRD standard decoction have been systematically described to provide a valuable reference for its clinical application and secondary development in the future, as well as some ideas for designing new preparations of classic TCM formulas.

## Supplementary Information

**Additional file 1.** Additional figures and tables.

## Data Availability

The raw data supporting the conclusions of this manuscript will be made available by the authors, without undue reservation, to any qualified researcher.

## Abbreviations

ACTH: Adrenocorticotropic hormone
CAM: Complementary and alternative medicines;
CMHS: China mental health survey
CRH: Corticotropin-releasing hormone
CORT: Corticosterone
CUMS: Chronic unpredictable mild stress
DA: Dopamine
DEG: Differentially expressed gene
ELISA: Enzyme linked immunosorbent assay
EPMT: Elevated plus maze test
FC: Fold change
FDR: False discovery rate
FST: Forced swimming test
GABA: γ-Aminobutyric acid
Glu: Glutamate
5-HT: 5-Hydroxytryptamine
HPA: Hypothalamic pituitary adrenocortical axis
HPLC: High performance liquid chromatography
IL: Interleukin
KEGG: Kyoto encyclopedia of genes and genomes
LBRD: Lily bulb and Rehmannia decoction
LFB: Luxol fast blue
mPFC: Medial prefrontal cortex
NE: Norepinephrine
OFT: Open field test
RIP: RNA immunoprecipitation
qRT-PCR: Quantitative RT-PCR
SD: Sprague–dawley
rTMS: Repetitive transcranial magnetic stimulation
SEM: Standard error of mean
SPF: Specific pathogen-free
SPT: Sucrose preference test
TCM: Traditional Chinese medicine
TNF-α: Tumor necrosis factor-α
TSH: Thyroid stimulating hormone
UHPLC-Q-TOF/MS: Utilized ultra-high performance liquid chromatography quadrupole time of flight tandem mass spectrometry
WB: Western blot

## References

[1] Menard C, Hodes GE, Russo SJ (2016). Pathogenesis of depression: Insights from human and rodent studies. Neuroscience.

[2] Huang Y, Wang Y, Wang H, Liu Z, Yu X, Yan J, Yu Y, Kou C, Xu X, Lu J, Wang Z, He S, Xu Y, He Y, Li T, Guo W, Tian H, Xu G, Xu X, Ma Y, Wang L, Wang L, Yan Y, Wang B, Xiao S, Zhou L, Li L, Tan L, Zhang T, Ma C, Li Q, Ding H, Geng H, Jia F, Shi J, Wang S, Zhang N, Du X, Du X, Wu Y (2019). Prevalence of mental disorders in China: a cross-sectional epidemiological study. Lancet Psychiatry.

[3] Filatova EV, Shadrina MI, Slominsky PA (2021). Major depression: one brain, one disease one set of intertwined processes. Cells..

[4] Bittar TP, Labonte B (2021). Functional contribution of the medial prefrontal circuitry in major depressive disorder and stress-induced depressive-like behaviors. Front Behav Neurosci.

[5] Chi X, Wang S, Baloch Z, Zhang H, Li X, Zhang Z, Zhang H, Dong Z, Lu Y, Yu H, Ma K (2019). Research progress on classical traditional Chinese medicine formula Lily Bulb and Rehmannia Decoction in the treatment of depression. Biomed Pharmacother.

[6] Lu Y, An T, Tian H, Gao X, Wang F, Wang S, Ma K (2020). Depression with comorbid diabetes: what evidence exists for treatments using traditional chinese medicine and natural products?. Front Pharmacol.

[7] Ma K, Wang X, Feng S, Xia X, Zhang H, Rahaman A, Dong Z, Lu Y, Li X, Zhou X, Zhao H, Wang Y, Wang S, Baloch Z (2020). From the perspective of Traditional Chinese Medicine: Treatment of mental disorders in COVID-19 survivors. Biomed Pharmacother.

[8] Shang B, Zhang H, Lu Y, Zhou X, Wang Y, Ma M, Ma K (2020). Insights from the perspective of traditional chinese medicine to elucidate association of lily disease and yin deficiency and internal heat of depression. Evid Based Complement Altern Med.

[9] Zhang H, Chi X, Pan W, Wang S, Zhang Z, Zhao H, Wang Y, Wu Z, Zhou M, Ma S, Zhao Q, Ma K (2020). Antidepressant mechanism of classical herbal formula lily bulb and Rehmannia decoction: insights from gene expression profile of medial prefrontal cortex of mice with stress-induced depression-like behavior. Genes Brain Behav..

[10] Guo T, Guo Y, Liu Q, Xu Y, Wei L, Wang Z, Chen S, Wang C, Tian Y, Cui J, Wang Y, Wang Y, Sun L (2021). The TCM prescription Ma-xing-shi-gan-tang inhibits *Streptococcus pneumoniae* pathogenesis by targeting pneumolysin. J Ethnopharmacol..

[11] Lin AX, Chan G, Hu Y, Ouyang D, Ung COL, Shi L, Hu H (2018). Internationalization of traditional Chinese medicine: current international market, internationalization challenges and prospective suggestions. Chin Med.

[12] Guo J, Zhang L, Shang Y, Yang X, Li J, He J, Gao X, Chang YX (2021). A strategy for intelligent chemical profiling-guided precise quantitation of multi-components in traditional Chinese medicine formulae-QiangHuoShengShi decoction. J Chromatogr A..

[13] Penner-Goeke S, Binder EB (2019). Epigenetics and depression. Dialogues Clin Neurosci.

[14] Lin E, Tsai SJ (2019). Epigenetics and depression: an update. Psychiatry Investig.

[15] Ma K, Xu A, Cui S, Sun MR, Xue YC, Wang JH (2016). Impaired GABA synthesis, uptake and release are associated with depression-like behaviors induced by chronic mild stress. Transl Psych..

[16] Ma K, Zhang H, Wang S, Wang H, Wang Y, Liu J, Song X, Dong Z, Han X, Zhang Y, Li H, Rahaman A, Wang S, Baloch Z (2019). The molecular mechanism underlying GABAergic dysfunction in nucleus accumbens of depression-like behaviours in mice. J Cell Mol Med.

[17] Ma K, Guo L, Xu A, Cui S, Wang JH (2016). Molecular Mechanism for Stress-Induced Depression Assessed by Sequencing miRNA and mRNA in Medial Prefrontal Cortex. PLoS ONE..

[18] Xu A, Cui S, Wang JH (2016). Incoordination among Subcellular Compartments Is Associated with Depression-Like Behavior Induced by Chronic Mild Stress. Int J Neuropsychopharmacol.

[19] Wang G, Wang C, Chen H, Chen L, Li J (2021). Activation of 6–8-week-old new mature adult-born dentate granule cells contributes to anxiety-like behavior. Neurobiol Stress..

[20] Liu S, Guo R, Liu F, Yuan Q, Yu Y, Ren F (2020). Gut microbiota regulates depression-like behavior in rats through the neuroendocrine-immune-mitochondrial pathway. Neuropsychiatr Dis Treat.

[21] Law CW, Chen Y, Shi W, Smyth GK (2014). voom: Precision weights unlock linear model analysis tools for RNA-seq read counts. Genome Biol.

[22] Ritchie ME, Phipson B, Wu D, Hu Y, Law CW, Shi W, Smyth GK (2015). limma powers differential expression analyses for RNA-sequencing and microarray studies. Nucleic acids research..

[23] Chen Z, Wang S, Li L, Huang Z, Ma K (2018). Anti-inflammatory effect of IL-37-Producing T-cell population in DSS-induced chronic inflammatory bowel disease in mice. Int J Mol Sci.

[24] Ren W, Liang P, Ma Y, Sun Q, Pu Q, Dong L, Luo G, Mazhar M, Liu J, Wang R, Yang S (2021). Research progress of traditional Chinese medicine against COVID-19. Biomed Pharmacother..

[25] Chu L, Huang F, Zhang M, Huang B, Wang Y (2021). Current status of traditional Chinese medicine for the treatment of COVID-19 in China. Chin Med.

[26] Zhao Z, Li Y, Zhou L, Zhou X, Xie B, Zhang W, Sun J (2021). Prevention and treatment of COVID-19 using traditional chinese medicine: a review. Phytomedicine..

[27] Xia Y, Shi LS, Chang JH, Miao HZ, Wang D (2021). Impact of the COVID-19 pandemic on intention to use traditional Chinese medicine: a cross-sectional study based on the theory of planned behavior. J Integr Med.

[28] Zhang W, Xie Q, Xu X, Sun S, Fan T, Wu X, Qu Y, Che J, Huang T, Li H, Zheng Y, Jiang C, Fang B, Zhou S (2021). Baidu Jieduan granules, traditional Chinese medicine, in the treatment of moderate coronavirus disease-2019 (COVID-19): study protocol for an open-label, randomized controlled clinical trial. Trials.

[29] Zheng Y (2021). Understanding COVID-19 in Wuhan from the perspective of cold-dampness: clinical evidences and mechanisms. Front Med..

[30] Luan X, Zhang LJ, Li XQ, Rahman K, Zhang H, Chen HZ, Zhang WD (2020). Compound-based Chinese medicine formula: From discovery to compatibility mechanism. J Ethnopharmacol..

[31] Zhou X, Li CG, Chang D, Bensoussan A (2019). Current status and major challenges to the safety and efficacy presented by chinese herbal medicine. Medicines..

[32] Chen ML, Gao J, He XR, Chen Q (2012). Involvement of the cerebral monoamine neurotransmitters system in antidepressant-like effects of a chinese herbal decoction, baihe dihuang tang, in mice model. Evid Based Compl Altern Med..

[33] Zhao HQ, Liu J, Meng P, Yang H, Lin XY, Long HP, Yu XM, Wang YH (2021). Effect of Baihe Dihuang Decoction on synaptic plasticity of hippocampus in rats with anxious depression. China J Chin Materia Medica..

[34] Tian JS, Meng Y, Wu YF, Zhao L, Xiang H, Jia JP, Qin XM (2019). A novel insight into the underlying mechanism of Baihe Dihuang Tang improving the state of psychological suboptimal health subjects obtained from plasma metabolic profiles and network analysis. J Pharm Biomed Anal.

[35] Sabti M, Sasaki K, Gadhi C, Isoda H (2019). Elucidation of the molecular mechanism underlying Lippia citriodora(Lim.)-induced relaxation and anti-depression. Int J Mol Sci..

[36] Lopez-Rodriguez R, Herrera-Ruiz M, Trejo-Tapia G, Dominguez-Mendoza BE, Gonzalez-Cortazar M, Zamilpa A (2019). In Vivo Gastroprotective and Antidepressant Effects of Iridoids, Verbascoside and Tenuifloroside from Castilleja tenuiflora Benth. Molecules..

[37] de Melo N, Sanchez-Ortiz BL, dos Sampaio TI, Pereira AC, da Silva Neto FL, da Silva H, et al. Anxiolytic and antidepressant effects of the hydroethanolic extract from the leaves of Aloysia polystachya (Griseb.) Moldenke: a study on zebrafish (Danio rerio). Pharmaceuticals. 2019, 12: 3. 10.3390/ph12030106 PMC6789669 31373315

[38] Cipriani A, Furukawa TA, Salanti G, Chaimani A, Atkinson LZ, Ogawa Y, Leucht S, Ruhe HG, Turner EH, Higgins JPT, Egger M, Takeshima N, Hayasaka Y, Imai H, Shinohara K, Tajika A, Ioannidis JPA, Geddes JR (2018). Comparative efficacy and acceptability of 21 antidepressant drugs for the acute treatment of adults with major depressive disorder: a systematic review and network meta-analysis. Lancet.

[39] Zhou X, Teng T, Zhang Y, Del Giovane C, Furukawa TA, Weisz JR, Li X, Cuijpers P, Coghill D, Xiang Y, Hetrick SE, Leucht S, Qin M, Barth J, Ravindran AV, Yang L, Curry J, Fan L, Silva SG, Cipriani A, Xie P (2020). Comparative efficacy and acceptability of antidepressants, psychotherapies, and their combination for acute treatment of children and adolescents with depressive disorder: a systematic review and network meta-analysis. Lancet Psychiatry.

[40] Tomlinson A, Efthimiou O, Boaden K, New E, Mather S, Salanti G, Imai H, Ogawa Y, Tajika A, Kishimoto S, Kikuchi S, Chevance A, Furukawa TA, Cipriani A (2019). Side effect profile and comparative tolerability of 21 antidepressants in the acute treatment of major depression in adults: protocol for a network meta-analysis. Evid Based Ment Health.

[41] Szoke-Kovacs Z, More C, Szoke-Kovacs R, Mathe E, Frecska E. Selective Inhibition of the Serotonin Transporter in the Treatment of Depression: Sertraline, Fluoxetine and Citalopram. Neuropsychopharmacologia Hungarica : a Magyar. Pszichofarmakologiai Egyesulet lapja. 2020, 22(1): 4–15. 32329748

[42] Araj-Khodaei M, Noorbala AA, Yarani R, Emadi F, Emaratkar E, Faghihzadeh S, Parsian Z, Alijaniha F, Kamalinejad M, Naseri M (2020). A double-blind, randomized pilot study for comparison of *Melissa officinalis* L. and *Lavandula angustifolia* Mill with Fluoxetine for the treatment of depression. BMC Complement Med Ther..

[43] Wang YS, Shen CY, Jiang JG (2019). Antidepressant active ingredients from herbs and nutraceuticals used in TCM: pharmacological mechanisms and prospects for drug discovery. Pharmacol Res..

[44] Miao M, Peng M, Chen H, Liu B (2019). Effects of Baihe Dihuang powder on chronic stress depression rat models. Saudi J Biol Sci.

[45] Zhao L, Wu YF, Gao Y, Xiang H, Qin XM, Tian JS (2017). Intervention mechanism of psychological sub-health by Baihe Dihuang Tang based on network pharmacology. Yao Xue Xue Bao..

[46] Wu H, Liu R, Wang J, Li T, Sun Y, Feng X, Bi Y, Zhang C, Sun Y, Liquid chromatography-mass spectrometry in-depth analysis and in silico verification of the potential active ingredients of Baihe Dihuang decoction in vivo and in vitro. J Separ Sci. 2021. 10.1002/jssc.202100434 34473407

[47] Zhang W, Wang JL, Zhang ZJ, Peng HS, Zhan ZL, Yang HJ (2019). Herbal textual research on triditional Chinese medicine "Baihe"( Lilii Bulbus). China J Chin Materia Medica..

[48] Zhou W, Cheng X, Zhang Y (2016). Effect of Liuwei Dihuang decoction, a traditional Chinese medicinal prescription, on the neuroendocrine immunomodulation network. Pharmacol Ther.

[49] Liu C, Ma R, Wang L, Zhu R, Liu H, Guo Y, Zhao B, Zhao S, Tang J, Li Y, Niu J, Fu M, Zhang D, Gao S (2017). Rehmanniae Radix in osteoporosis: A review of traditional Chinese medicinal uses, phytochemistry, pharmacokinetics and pharmacology. J Ethnopharmacol.

[50] Dean J, Keshavan M (2017). The neurobiology of depression: an integrated view. Asian J Psychiatr.

[51] Maletic V, Robinson M, Oakes T, Iyengar S, Ball SG, Russell J (2007). Neurobiology of depression: an integrated view of key findings. Int J Clin Pract.

[52] Juruena MF, Eror F, Cleare AJ, Young AH (2020). The role of early life stress in HPA axis and anxiety. Adv Exp Med Biol.

[53] Dell'Osso L, Carmassi C, Mucci F, Marazziti D (2016). Depression, serotonin and tryptophan. Curr Pharm Des.

[54] Diederichs C, DeMayo MM, Cole J, Yatham LN, Harris AD, McGirr A (2021). Intermittent theta-burst stimulation transcranial magnetic stimulation Increases GABA in the medial prefrontal cortex: a preliminary sham-controlled magnetic resonance spectroscopy study in acute bipolar depression. Front Psychiatry..

[55] McCarty R, Josephs T, Kovtun O, Rosenthal SJ (2021). Correction: Enlightened: addressing circadian and seasonal changes in photoperiod in animal models of bipolar disorder. Transl Psychiatry.

[56] Pizzagalli DA, Roberts AC, Prefrontal cortex and depression. Neuropsychopharmacology : official publication of the American College of Neuropsychopharmacology. 2021. 10.1038/s41386-021-01101-7 PMC8617037 34341498

[57] Lee CW, Wu HF, Chu MC, Chung YJ, Mao WC, Li CT, Lin HC (2021). Mechanism of intermittent theta-burst stimulation in synaptic pathology in the prefrontal cortex in an antidepressant-resistant depression rat model. Cereb Cortex.

[58] Rajkowska G, Stockmeier CA (2013). Astrocyte pathology in major depressive disorder: insights from human postmortem brain tissue. Curr Drug Targets.

[59] Barnhofer T, Reess TJ, Fissler M, Winnebeck E, Grimm S, Gartner M, Fan Y, Huntenburg JM, Schroeter TA, Gummersbach M, Bajbouj M, Holzel BK (2021). Effects of mindfulness training on emotion regulation in patients with depression: reduced dorsolateral prefrontal cortex activation indexes early beneficial changes. Psychosom Med.

[60] Kajitani N, Iwata M, Miura A, Tsunetomi K, Yamanashi T, Matsuo R, Nishiguchi T, Fukuda S, Nagata M, Shibushita M, Yamauchi T, Pu S, Shirayama Y, Watanabe K, Kaneko K (2020). Prefrontal cortex infusion of beta-hydroxybutyrate, an endogenous NLRP3 inflammasome inhibitor, produces antidepressant-like effects in a rodent model of depression. Neuropsychopharmacol Rep.

[61] Lei Y, Wang J, Wang D, Li C, Liu B, Fang X, You J, Guo M, Lu XY (2020). SIRT1 in forebrain excitatory neurons produces sexually dimorphic effects on depression-related behaviors and modulates neuronal excitability and synaptic transmission in the medial prefrontal cortex. Mol Psychiatry.

[62] Price RB, Duman R (2020). Neuroplasticity in cognitive and psychological mechanisms of depression: an integrative model. Mol Psychiatry.

[63] Duman RS, Aghajanian GK (2012). Synaptic dysfunction in depression: potential therapeutic targets. Science.

[64] Seo JS, Wei J, Qin L, Kim Y, Yan Z, Greengard P (2017). Cellular and molecular basis for stress-induced depression. Mol Psychiatry.

[65] Zhou XT, Bao WD, Liu D, Zhu LQ (2020). Targeting the neuronal activity of prefrontal cortex: new directions for the therapy of depression. Curr Neuropharmacol.

[66] Park HS, Kim J, Ahn SH, Ryu HY (2021). Epigenetic targeting of histone deacetylases in diagnostics and treatment of depression. Int J Mol Sci.

[67] Poon CH, Heng BC, Lim LW (2021). New insights on brain-derived neurotrophic factor epigenetics: from depression to memory extinction. Ann N Y Acad Sci.

[68] Juruena MF, Gadelrab R, Cleare AJ, Young AH (2021). Epigenetics: a missing link between early life stress and depression. Progr Neuro-psychopharmacol Biol Psychiatry..

[69] Carnevali GS, Buoli M (2021). The role of epigenetics in perinatal depression: Are there any candidate biomarkers?. J Affect Disord.

[70] Allen L, Dwivedi Y (2020). MicroRNA mediators of early life stress vulnerability to depression and suicidal behavior. Mol Psychiatry.

[71] Khodadadegan MA, Azami S, Guest PC, Jamialahmadi T, Sahebkar A (2021). Effects of curcumin on depression and anxiety: a narrative review of the recent clinical data. Adv Exp Med Biol.

[72] Fee C, Banasr M, Sibille E (2017). Somatostatin-positive gamma-aminobutyric acid interneuron deficits in depression: cortical microcircuit and therapeutic perspectives. Biol Psychiat.

[73] Tao WW, Jiang H, Tao XM, Jiang P, Sha LY, Sun XC (2016). Effects of Acupuncture, Tuina, Tai Chi, Qigong, and traditional chinese medicine five-element music therapy on symptom management and quality of life for cancer patients: a meta-analysis. J Pain Symptom Manage.

[74] Alario AA, Niciu MJ (2021). Biomarkers of ketamine's antidepressant effect: a clinical review of genetics, functional connectivity, and neurophysiology. Chronic stress.

[75] Qiao X, Gai H, Su R, Deji C, Cui J, Lai J, Zhu Y (2018). PI3K-AKT-GSK3beta-CREB signaling pathway regulates anxiety-like behavior in rats following alcohol withdrawal. J Affect Disord.

[76] Babaev O, Piletti Chatain C, Krueger-Burg D (2018). Inhibition in the amygdala anxiety circuitry. Exp Mol Med.

[77] Tian JS, Liu SB, He XY, Xiang H, Chen JL, Gao Y, Zhou YZ, Qin XM (2018). Metabolomics studies on corticosterone-induced PC12 cells: A strategy for evaluating an in vitro depression model and revealing the metabolic regulation mechanism. Neurotoxicol Teratol.

[78] Ma RD, Zhou GJ, Qu M, Yi JH, Tang YL, Yang XY, Nie YX, Gu HF (2020). Corticosterone induces neurotoxicity in PC12 cells via disrupting autophagy flux mediated by AMPK/mTOR signaling. CNS Neurosci Ther.

[79] Oh H, Lewis DA, Sibille E (2016). The role of BDNF in age-dependent changes of excitatory and inhibitory synaptic markers in the human prefrontal cortex. Neuropsychopharmacology.

[80] Fogaca MV, Wu M, Li C, Li XY, Picciotto MR, Duman RS, Inhibition of GABA interneurons in the mPFC is sufficient and necessary for rapid antidepressant responses. Mol Psychiatry. 2020. 10.1038/s41380-020-00916-y PMC8052382 33070149

[81] Xiao Q, Zhou X, Wei P, Xie L, Han Y, Wang J, Cai A, Xu F, Tu J, Wang L, A new GABAergic somatostatin projection from the BNST onto accumbal parvalbumin neurons controls anxiety. Mol Psychiatry. 2020. 10.1038/s41380-020-0816-3 PMC8589681 32555286

[82] Marchisella F, Creutzberg KC, Begni V, Sanson A, Wearick-Silva LE, Tractenberg SG, Orso R, Kestering-Ferreira E, Grassi-Oliveira R, Riva MA (2021). Exposure to prenatal stress is associated with an excitatory/inhibitory imbalance in rat prefrontal cortex and amygdala and an increased risk for emotional dysregulation. Front Cell Dev Biol..

[83] Sarawagi A, Soni ND, Patel AB (2021). Glutamate and GABA homeostasis and neurometabolism in major depressive disorder. Front Psychiatry..

[84] Keshavarzi S, Kermanshahi S, Karami L, Motaghinejad M, Motevalian M, Sadr S (2019). Protective role of metformin against methamphetamine induced anxiety, depression, cognition impairment and neurodegeneration in rat: the role of CREB/BDNF and Akt/GSK3 signaling pathways. Neurotoxicology.

[85] Murphy CP, Li X, Maurer V, Oberhauser M, Gstir R, Wearick-Silva LE, Viola TW, Schafferer S, Grassi-Oliveira R, Whittle N, Huttenhofer A, Bredy TW, Singewald N (2017). MicroRNA-mediated rescue of fear extinction memory by miR-144-3p in extinction-impaired mice. Biol Psychiat.

[86] Jadeja RN, Jones MA, Abdelrahman AA, Powell FL, Thounaojam MC, Gutsaeva D, Bartoli M, Martin PM (2020). Inhibiting microRNA-144 potentiates Nrf2-dependent antioxidant signaling in RPE and protects against oxidative stress-induced outer retinal degeneration. Redox biology..

[87] Li Y, Zhao Y, Cheng M, Qiao Y, Wang Y, Xiong W, Yue W (2018). Suppression of microRNA-144–3p attenuates oxygen-glucose deprivation/reoxygenation-induced neuronal injury by promoting Brg1/Nrf2/ARE signaling. J Biochem Mol Toxicol..

[88] Zhou LT, Zhang J, Tan L, Huang HZ, Zhou Y, Liu ZQ, Lu Y, Zhu LQ, Yao C, Liu D (2021). Elevated levels of miR-144–3p induce cholinergic degeneration by impairing the maturation of NGF in Alzheimer's disease. Front Cell Devel Biol..

[89] Blazon M, LaCarubba B, Bunda A, Czepiel N, Mallat S, Londrigan L, Andrade A (2021). N-type calcium channels control GABAergic transmission in brain areas related to fear and anxiety. OBM Neurobiol..

[90] Jiang H, Liu JP, Xi K, Liu LY, Kong LY, Cai J, Cai SQ, Han XY, Song JG, Yang XM, Wan Y, Xing GG (2021). Contribution of AMPA receptor-mediated LTD in LA/BLA-CeA pathway to comorbid aversive and depressive symptoms in neuropathic pain. J Neurosci.

[91] Bastle RM, Oliver RJ, Gardiner AS, Pentkowski NS, Bolognani F, Allan AM, Chaudhury T, St Peter M, Galles N, Smith C, Neisewander JL, Perrone-Bizzozero NI (2018). In silico identification and in vivo validation of miR-495 as a novel regulator of motivation for cocaine that targets multiple addiction-related networks in the nucleus accumbens. Mol Psychiatry.

[92] Kim J, Lee S, Kang S, Kim SH, Kim JC, Yang M, Moon C (2017). Brain-derived neurotropic factor and GABAergic transmission in neurodegeneration and neuroregeneration. Neural Regen Res.

[93] Tomoda T, Sumitomo A, Shukla R, Hirota-Tsuyada Y, Miyachi H, Oh H, French L, Sibille E, BDNF controls GABAAR trafficking and related cognitive processes via autophagic regulation of p62. Neuropsychopharmacology. 2021. 10.1038/s41386-021-01116-0 PMC8674239 34341497

