# Supplementary material for: Integrated analysis of the chemical-material basis and molecular mechanisms for the classic herbal formula of Lily Bulb and Rehmannia Decoction in alleviating depression
Source: Chin Med. 2021 Oct 21;16:107. doi: 10.1186/s13020-021-00519-x (PMC8529377; doi:10.1186/s13020-021-00519-x)
Supplement: Supplementary file 1 — Additional file 1. Additional figures and tables. [file 13020_2021_519_MOESM1_ESM.docx]

**Integrated analysis of the chemical-material basis and molecular mechanisms for classical herbal formula Lily Bulb and Rehmannia Decoction alleviating depression**

Hongxiu Zhang ^1,2 #^, Xiaoyan Xue ^1 #^, Jin Pan ^1, #^, Xiaobin Song ^1 #^, Xing Chang ^3^, Qiancheng Mao ^1^, Yanting Lu ^1^, Haijun Zhao ^1^, Yuan Wang ^1^, Xiansu Chi ^4^, Shijun Wang ^1^ * and Ke Ma ^1^ *

*1 Shandong Co-Innovation Center of Classic TCM Formula, Shandong University of Traditional Chinese Medicine, Jinan 250355, PR China*

*2 Institute of Virology, Jinan Municipal Center for Disease Control and Prevention, Jinan 250021, PR China*

*3 Department of Cardiology, Guang'anmen Hospital, Chinese Academy of Traditional Chinese Medicine, Beijing 100053, PR China*

*4 Department of brain disease, Xiyuan Hospital, Chinese Academy of Traditional Chinese Medicine, Beijing 100091, PR China*

**Running title:** miRNA/mRNA regulatory network of LBRD treatment depression

# These authors contributed equally to this work

**Address Correspondence to:**

Dr. Ke Ma, Shandong Co-Innovation Center of Classic TCM Formula, Shandong University of Traditional Chinese Medicine, No 4655, University Road, Changqing District, Jinan, Shandong 250355, People’s Republic of China, Tel/Fax +86-531-89628077, Email: make19880710@163.com.

Or Dr. Shijun Wang, Shandong Co-Innovation Center of Classic TCM formula, Shandong University of Traditional Chinese Medicine, No. 4655, University Road, Changqing District, Jinan, Shandong 250355, P.R. China, Tel/Fax: 86-531-89628750, E-mail: [pathology@163.com](mailto:pathology@163.com).

**Table S1: Chronic unpredictable mild stress procedure**

| Stressor | **Mon** | **Tue** | **Wed** | **Thu** | **Fri** | **Sat** | **Sun** |
| --- | --- | --- | --- | --- | --- | --- | --- |
| Food and wather deprivation | 09:00→09:00  (24 h) |  |  |  |  |  |  |
| Exposure to empty bottles |  | 9:00-11:00 |  |  |  |  |  |
| Soiled cage |  | 11:00→11:00  (24 h) |  |  | 9:00-21:00 |  |  |
| Restraint |  |  | 11:00-12:00 |  |  |  |  |
| Light/dark succession every 2 h |  |  | 12:00-22:00 |  |  |  |  |
| 45°cage title |  |  |  | 9:00-21:00 |  |  |  |
| Stroboscope |  |  |  | 21:00→9:00  (12 h) |  |  |  |
| Cold  (4°for 1h) |  |  |  |  | 21:00-22:00 |  |  |
| Cage rotation | 11:00-12:00 |  |  |  |  | 9:00-10:00 |  |
| Wet cage |  |  |  |  |  | 10:00-22:00 |  |
| White noise |  |  |  |  |  |  | 9:00-21:00 |
| Space reduction |  |  |  |  |  |  | 21:00→ |

**Table S2: The elution gradient of lily bulb decoction, fresh raw Rehmannia root and LBRD standard decoction by UHPLC-Q-TOF/MS.**

| **Time (min)** | 0.01 | 2 | 4 | 8 | 10 | 14 | 15 | 15.1 | 16 |
| --- | --- | --- | --- | --- | --- | --- | --- | --- | --- |
| **A%** | 95 | 95 | 70 | 50 | 20 | 0 | 0 | 95 | 95 |
| **B%** | 5 | 5 | 30 | 50 | 80 | 100 | 100 | 5 | 5 |

**Table S3: The specific mass parameters of LBRD standard decoction by UHPLC-Q-TOF/MS.**

| Mass parameters | Nebulizer Gas (GS1, PSI) | Auxiliary Gas (GS2, PSI) | Curtain Gas (CUR, PSI） | Ion Source Temperature (℃) | Ion Spray Voltage (V) | Declustering Potential (DP ,V) | Mass Scan Range (TOF MS scan) | Collision Energy （TOF MS scan, eV） | Mass Scan Range (Product Ion scan) | Collision Energy (Product Ion scan,eV） | Interface Heater Temperature (℃) |
| --- | --- | --- | --- | --- | --- | --- | --- | --- | --- | --- | --- |
| Positive ion | 55 | 55 | 35 | 550 | 5500 | 80 | 100-1000 | 10 | 40-1000 | 35 | 550 |
| Negative ion | 55 | 55 | 35 | 550 | -4500 | -80 | 100-1000 | -10 | 40-1000 | -35 | 550 |

**Table S4: Quality control of verbascoside in LBRD standard decoction by HPLC analysis**

| **Ingredients** | **Retention time (s)** | **Area** | **High** | **Concentration** | **Percentage (W/W)** |
| --- | --- | --- | --- | --- | --- |
| Verbascoside Standard | 16.412 | 92393 | 6331 | 99.6mg/L |  |
| Fresh lily bulb decoction | 16.757 | 36859 | 2669 | 15.8 mg/L | 0.001264% |
| Fresh raw Rehmannia juice | 16.762 | 64621 | 5401 | 27.67 mg/L | 0.002767% |
| LBRD standard decoction | 16.762 | 317728 | 23330 | 135.7 mg/L | 0.009046% |

**Table S5: The changed miRNAs predict target mRNAs under LBRD standard decoction treatment of CUMS-induced rats depression.**

| **mRNAs** | **miRNAs that are predicted to mRNAs by Targetscan, RNA22 and miRDB** |
| --- | --- |
| Btg2 ↑ | rno-miR-21-5p↓, rno-miR-25-3p↓ |
| Slc39a12↑ | rno-miR-7a-5p↓ |
| Calml4↑ | rno-miR-144-3p↓, rno-miR-151-3p↓, rno-miR-204-5p↓, rno-miR-495↓, rno-miR-499-5p↓, rno-miR-879-5p↓ |
| Foxs1↑ | rno-miR-199a-3p↓ |
| Nr1d1↑ | rno-miR-24-3p↓ |
| Ntrk2↑ | rno-miR-144-3p↓, rno-miR-151-3p↓, rno-miR-199a-3p↓, rno-miR-204-5p↓, no-miR-7a-5p↓ |
| Tnfaip6↑ | rno-miR-495↓ |
| Arc↑ | rno-miR-495↓, rno-miR-879-5p↓ |
| Ntf3↑ | rno-miR-21-5p↓ |
| GAT-3↑ | rno-miR-25-3p↓, rno-miR-495↓, rno-miR-7a-5p↓ |
| VGAT↑ | rno-miR-144-3p↓, rno-miR-204-5p↓, rno-miR-25-3p↓ |
| Gad1↑ | rno-miR-144-3p↓, rno-miR-495↓, rno-miR-879-5p↓ |
| Bdnf↑ | rno-miR-495↓ |
| Dbp↓ | rno-miR-206-3p↑, rno-miR-346↑ |
| Flot2↓ | rno-miR-31a-5p↑, rno-miR-34c-5p↑ |
| Acvr1c↓ | rno-miR-206-3p↑, rno-miR-31a-5p↑, rno-miR-346↑, rno-miR-34c-5p↑, rno-miR-380-5p↑, rno-miR-494-3p↑ |
| Syt6↓ | rno-miR-31a-5p↑, rno-miR-34c-5p↑, rno-miR-365-3p↑, rno-miR-494-3p↑ |
| Il18bp↓ | rno-miR-34c-5p↑ |

**Table S6: Signaling pathways identified by KEGG function analysis based on miRNA**

**predicted target mRNAs overlapped DEGs in rats mPFC transcriptome**

| **Pathway** | **DEGs with pathway annotation (7)** | **All genes with pathway annotation (8888)** | **Contributed Genes** | **P-value** | **KEGG Pathway Term ID** |
| --- | --- | --- | --- | --- | --- |
| Neurotrophin signaling pathway | (4) 57.14% | (133) 1.50% | Bdnf, Ntrk2, Calml4, Ntf3 | 1.62E-06 | 4722 |
| Ras signaling pathway | (4) 57.14% | (283) 3.18% | Bdnf, Ntrk2, Calml4, Ntf4 | 3.27E-05 | 4014 |
| MAPK signaling pathway | (3) 42.86% | (335) 3.8% | Bdnf, Ntrk2, Ntf3 | 0.001658812 | 4010 |
| PI3K-Akt signaling pathway | (3) 42.86% | (396) 4.46% | Bdnf, Ntrk2, Ntf4 | 0.002686471 | 4151 |
| Quorum sensing | (1) 14.29% | (9) 0.10% | Gad1 | 0.007069092 | 2024 |
| Taurine and hypotaurine metabolism | (1) 14.29% | (11) 0.12% | Gad1 | 0.008634171 | 430 |
| cAMP signaling pathway | (2) 28.57% | (204) 2.30% | Bdnf, Calml4 | 0.0102035 | 4024 |
| MAPK signaling pathway - plant | (1) 14.29% | (16) 0.18% | Calml4 | 0.01253762 | 4016 |
| Plant-pathogen interaction | (1) 14.29% | (19) 0.21% | Calml4 | 0.01487336 | 4626 |
| Alanine, aspartate and glutamate metabolism | (1) 14.29% | (38) 0.43% | Gad1 | 0.0295567 | 250 |
| beta-Alanine metabolism | (1) 14.29% | (36) 0.41% | Gad1 | 0.02801997 | 410 |
| Butanoate metabolism | (1) 14.29% | (30) 0.34% | Gad1 | 0.02339727 | 650 |
| Circadian rhythm | (1) 14.29% | (31) 0.35% | Nr1d1 | 0.02416903 | 4710 |
| Phototransduction | (1) 14.29% | (27) 0.30% | Calml4 | 0.02107886 | 4744 |
| Phototransduction - fly | (1) 14.29% | (33) 0.37% | Calml4 | 0.02571097 | 4745 |
| RNA degradation | (1) 14.29% | (96) 1.08% | Btg2 | 0.02922518 | 3018 |
| Phosphatidylinositol signaling system | (1) 14.29% | (101) 1.14% | Calml4 | 0.03690954 | 4070 |
| Circadian entrainment | (1) 14.29% | (101) 1.14% | Calml4 | 0.03990954 | 4713 |
| Long-term potentiation | (1) 14.29% | (71) 0.80% | Calml4 | 0.04461372 | 4720 |
| GABAergic synapse | (1) 14.29% | (97) 1.09% | Gad1 | 0.04739636 | 4727 |

**Table S7: Signaling pathways identified by KEGG function analysis based on DEGs data after LBRD standard decoction-containing serum administration of PC12 cells**

| **Pathway** | | **DEGs with pathway annotation (47)** | **All genes with pathway annotation (8888)** | **Contributed Genes** | **P-value** | **KEGG Pathway ID** |
| --- | --- | --- | --- | --- | --- | --- |
| Ras signaling pathway | （7）15.91% | | （283）3.18% | Bdnf,Ntrk2,Ntf4,Calml3,Calml4,Gng13,Lat | 6.50E-04 | 4014 |
| Neurotrophin signaling pathway | （5）11.36% | | （133）1.50% | Bdnf,Ntrk2,Ntf4,Calml3,Calml4 | 6.45E-04 | 4722 |
| Phototransduction - fly | （3）6.82% | | （33）0.371% | Calml3,Gng13,Calml4 | 6.77E-04 | 4745 |
| Circadian entrainment | （4）9.09% | | （101）1.14% | Calml3,Fos,Gng13,Calml4 | 0.00192628 | 4713 |
| MAPK signaling pathway - plant | （2）4.55% | | （16）0.18% | Calml3,Calml4 | 0.003133008 | 4016 |
| cAMP signaling pathway | （5）11.36% | | （204）2.30% | Bdnf,Amh,Calml3,Fos,Calml4 | 0.004252543 | 4024 |
| Plant-pathogen interaction | （2）4.55% | | （19）0.21% | Calml3,Calml4 | 0.004419702 | 4626 |
| Inflammatory mediator regulation of TRP channels | （4）9.09% | | （124）1.40% | Asic3,Htr2b,Calml3,Calml4 | 0.004051236 | 4750 |
| Dopaminergic synapse | （4）9.09% | | （142）1.60% | Calml3,Fos,Gng13,Calml4 | 0.006542667 | 4728 |
| Relaxin signaling pathway | （4）9.09% | | （144）1.62% | Rxfp1,Fos,Gng13,Col3a1 | 0.006870355 | 4926 |
| Phototransduction | （2）4.55% | | （27）0.30% | Calml3,Calml4 | 0.008831556 | 4744 |
| Butanoate metabolism | （2）4.55% | | （30）0.34% | Gad1,Acsm3 | 0.0108356 | 650 |
| Circadian rhythm | （2）4.55% | | （31）0.35% | Nr1d1,Npas2 | 0.01154415 | 4710 |
| GnRH signaling pathway | （3）6.82% | | （100）1.13% | Calml3,Ptk2b,Calml4 | 0.01566847 | 4912 |
| Glucagon signaling pathway | （3）6.82% | | （109）1.23% | Pgam2,Calml3,Calml4 | 0.01968389 | 4922 |
| Calcium signaling pathway | （4）9.09% | | （214）2.41% | Htr2b,Calml3,Ptk2b,Calml4 | 0.02600306 | 4020 |
| Neuroactive ligand-receptor interaction | （5）11.36% | | （316）3.56% | Ghrhr,Rxfp1,Htr2b,S1pr3,Uts2r | 0.02490304 | 4080 |
| C-type lectin receptor signaling pathway | （3）6.82% | | （124）1.40% | Clec4m,Calml3,Calml4 | 0.02752997 | 4625 |
| Taurine and hypotaurine metabolism | （1）2.27% | | (11)12% | Gad1 | 0.05668554 | 430 |
| Quorum sensing | （1）2.27% | | （9）0.10% | Gad1 | 0.04661845 | 2024 |

**Table S8: The changed miRNAs predict target mRNAs after LBRD standard decoction-containing serum administration of CORT-induced PC12 cells depression model.**

| **mRNAs** | **miRNAs that are predicted to mRNAs by Targetscan, RNA22 and miRDB** |
| --- | --- |
| Mbp↑ | rno-miR-125b-5p↓，rno-miR-7a-2-3p↓，rno-miR-130b-5p↓，rno-miR-532-3p↓ |
| Gad1↑ | rno-miR-495↓，rno-miR-130b-5p↓，rno-miR-144-3p↓ |
| Bdnf↑ | rno-miR-495↓，rno-miR-125b-5p↓，rno-miR-336-5p↓，rno-miR-7a-2-3p↓，rno-miR-130b-5p↓ |
| Ntrk2↑ | rno-miR-144-3p↓，rno-miR-125b-5p↓，rno-miR-130b-5p↓，rno-miR-23a-5p↓ |
| Enpep↑ | rno-miR-125b-5p↓，rno-miR-130b-5p↓ |
| Mefv↑ | rno-miR-336-5p↓，rno-miR-532-3p↓，rno-miR-144-3p↓ |
| VGAT ↑ | rno-miR-144-3p↓，rno-miR-23a-5p↓ |
| Pex12↑ | rno-miR-495↓ |
| Wipf3↑ | rno-miR-125b-5p↓，rno-miR-130b-5p↓，rno-miR-495↓，rno-miR-144-3p↓ |
| Nr1d1↑ | rno-miR-24-3p↓ |
| Itgb6↑ | rno-miR-125b-5p↓ |
| Calml3↑ | rno-miR-495↓ |
| S1pr3↑ | rno-miR-125b-5p↓，rno-miR-495↓ |
| Psd3↑ | rno-miR-23a-5p↓，rno-miR-144-3p↓，rno-miR-336-5p↓，rno-miR-495↓，rno-miR-532-3p↓ |
| Calml4↑ | rno-miR-144-3p↓，rno-miR-495↓，rno-miR-125b-5p↓，rno-miR-336-5p↓，rno-miR-7a-2-3p↓，rno-miR-130b-5p↓ |
| Npas2↑ | rno-miR-130b-5p↓，rno-miR-532-3p↓，rno-miR-495↓ |
| Zswim5↓ | rno-let-7b-3p↑，rno-miR-708-5p↑ |
| Crb3↓ | rno-miR-708-5p↑，rno-miR-331-3p↑ |
| Lifr↓ | rno-let-7b-3p↑，rno-miR-708-5p↑ |
| Ghrhr↓ | rno-miR-34c-5p↑ |

**Table S9: Signaling pathways identified by KEGG function analysis based on miRNA predicted target mRNAs overlapped DEGs in cell transcriptome**

| **Pathway** | **DEGs with pathway annotation (18)** | **All genes with pathway annotation (8888)** | **Contributed Genes** | **P-value** | **KEGG Pathway ID** |
| --- | --- | --- | --- | --- | --- |
| Neurotrophin signaling pathway | (4)22.22% | (133)1.50% | Bdnf,Ntrk2, Calml3,Calml4 | 1.25E-04 | 4722 |
| MAPK signaling pathway | (2)11.11% | (16)0.18% | Calml3,Calml4 | 4.57E-04 | 4016 |
| Plant-pathogen interaction | (2)11.11% | (19)0.21% | Calml3,Calml4 | 6.49E-04 | 4626 |
| Circadian rhythm | (2)11.11% | (31)0.35% | Nr1d1,Npas2 | 0.00173981 | 4710 |
| Phototransduction | (2)11.11% | (27)0.30% | Calml3,Calml4 | 0.001319589 | 4744 |
| Phototransduction | (2)11.11% | (33)0.37% | Calml3,Calml4 | 0.001970796 | 4745 |
| Ras signaling pathway | (4)22.22% | (283)3.18% | Bdnf,Ntrk2, Calml3,Calml4 | 0.002162797 | 4014 |
| cAMP signaling pathway | (3)16.67% | (204)2.30% | Bdnf,Calml3,Calml4 | 0.007538358 | 4024 |
| Long-term potentiation | (2)11.11% | (71)0.80% | Calml3,Calml4 | 0.008862956 | 4720 |
| Renin secretion | (2)11.11% | (70)0.79% | Calml3,Calml4 | 0.008623599 | 4924 |
| Gastric acid secretion | (2)11.11% | (77)0.87% | Calml3,Calml4 | 0.01036125 | 4971 |
| Quorum sensing | (1)5.56% | (9)0.10% | Gad1 | 0.01808794 | 2024 |
| Phosphatidylinositol signaling system | (2)11.11% | (101)1.136% | Calml3,Calml4 | 0.01737741 | 4070 |
| Circadian entrainment | (2)11.11% | (101)1.136% | Calml3,Calml4 | 0.01737741 | 4713 |
| GABAergic synapse | (2)11.11% | (97)1.09% | Gad1,Vgat | 0.01786564 | 4727 |
| GnRH signaling pathway | (2)11.11% | (100)1.13% | Calml3,Calml4 | 0.01705364 | 4912 |
| Melanogenesis | (2)11.11% | (105)1.18% | Calml3,Calml4 | 0.01869883 | 4916 |
| Aldosterone synthesis and secretion | (2)11.11% | (104)1.17% | Calml3,Calml4 | 0.01836455 | 4925 |
| Salivary secretion | (2)11.11% | (88)0.99% | Calml3,Calml4 | 0.01337838 | 4970 |
| Taurine and hypotaurine metabolism | (1)5.56% | (11)0.12% | Gad1 | 0.0220653 | 430 |

**Figure S1: Representative base peak chromatogram of fresh lily bulb decoction and fresh Rehmannia juice in positive and negative ionization mode, respectively.**

**
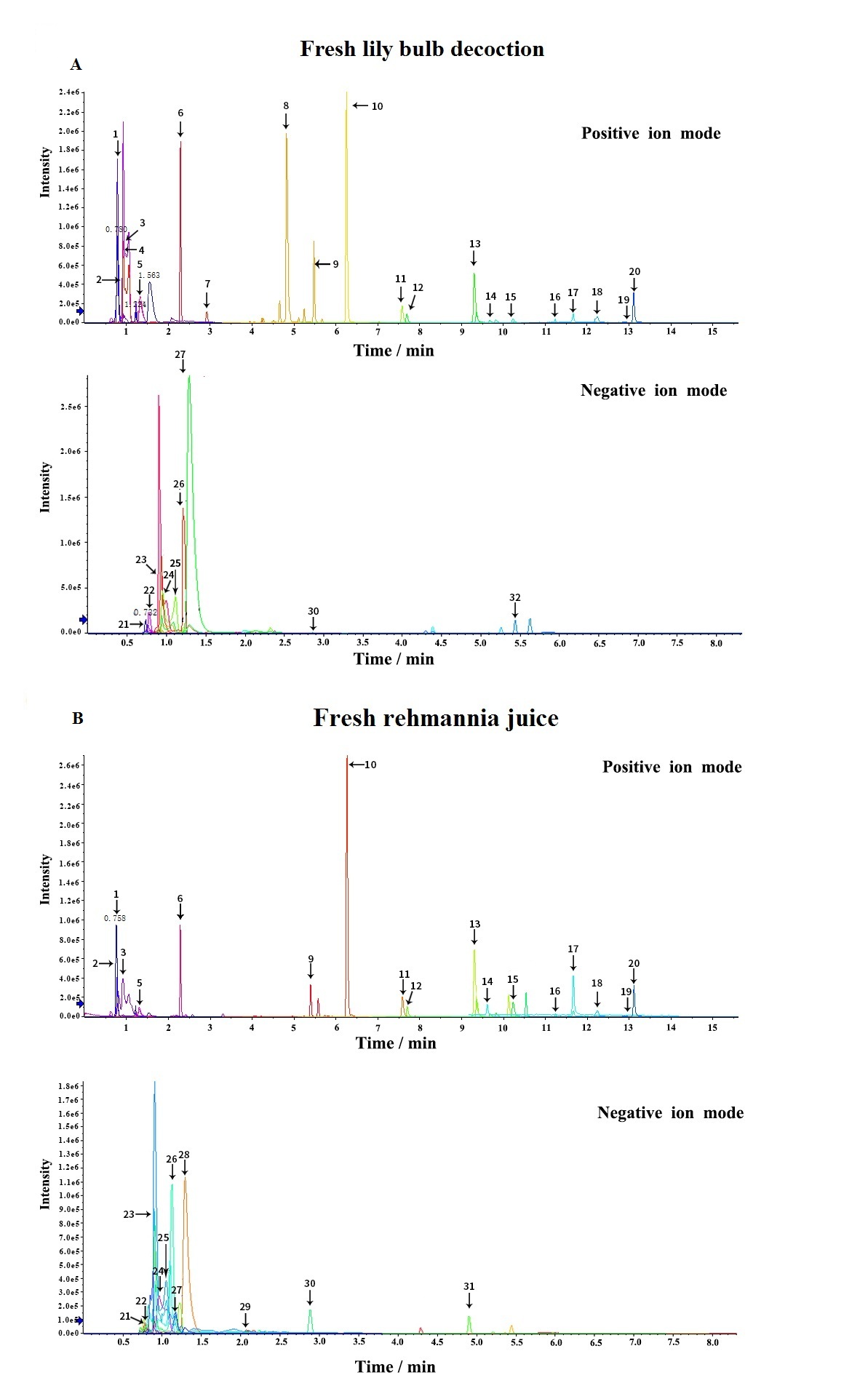
**

**Figure S2: HPLC chromatograms of verbascoside in the** fresh lily bulb decoction**, fresh Rehmannia juice and LBRD standard decoction.** The quality of marker content in the fresh lily bulb decoction, fresh Rehmannia juice and LBRD standard decoction were measured by Waters XBridge C18 reversed-phase column with use the verbascoside standard (A). White arrow indicates the HPLC chromatogram of verbascoside in fresh lily bulb decoction (B) , fresh Rehmannia juice (C) and LBRD standard decoction (D).

**
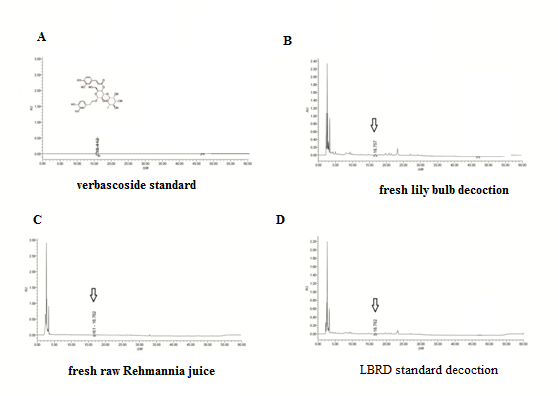
**

**Figure S3: KEGG pathway of LBRD standard decoction-containing serum treatment of CORT-induced cell depression model.**

**
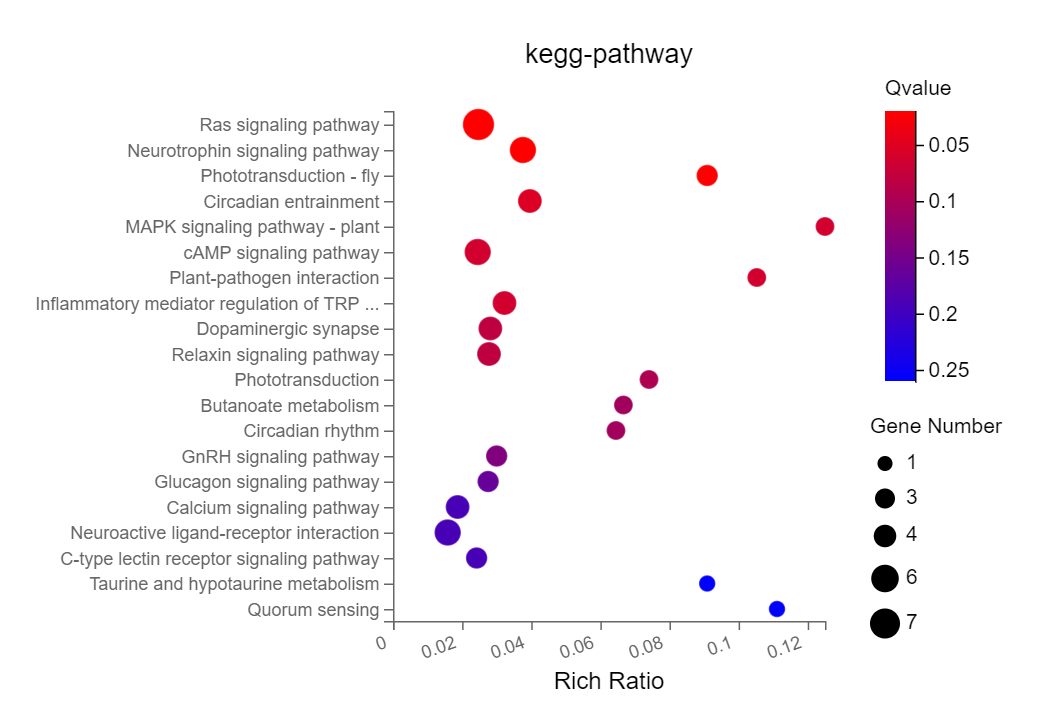
**

**Figure S4: Verbascoside attenuated the depressive symptoms in chronic stress exposed rats.** The percent of sucrose consumption/total water in the SPT. Total time spent immobile in TST. Active time of rats in the center of OFT. Percentage of active time in the open arms of EPMT. All data are presented as mean ± SEM (n=12 per group), ^***^*P*<0.001 compare to control group; ^##^*P*<0.01, ^###^*P*<0.001 compare to CUMS+Saline group. CUMS+Saline: CUMS+Saline group, CUMS+LBRD: CUMS+Lily Bulb and Rehmannia Decoction group, CUMS+Verb: CUMS+ Verbascoside group, CUMS+Flu: CUMS+fluoxetine group,

**
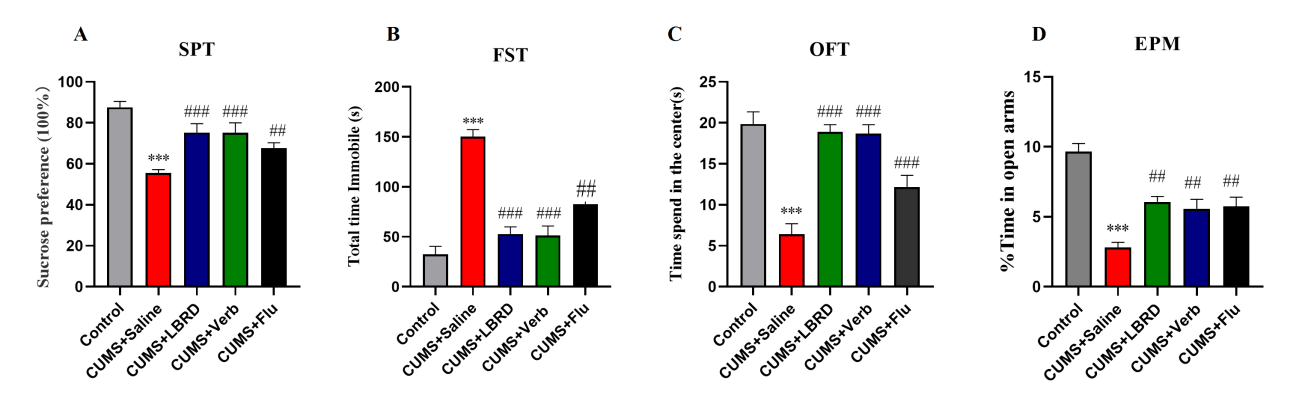
**

**Figure S5: Establishment and evaluation of depression model of yin-deficiency and inner-heat type with emotional symptoms of lily disease.**

**
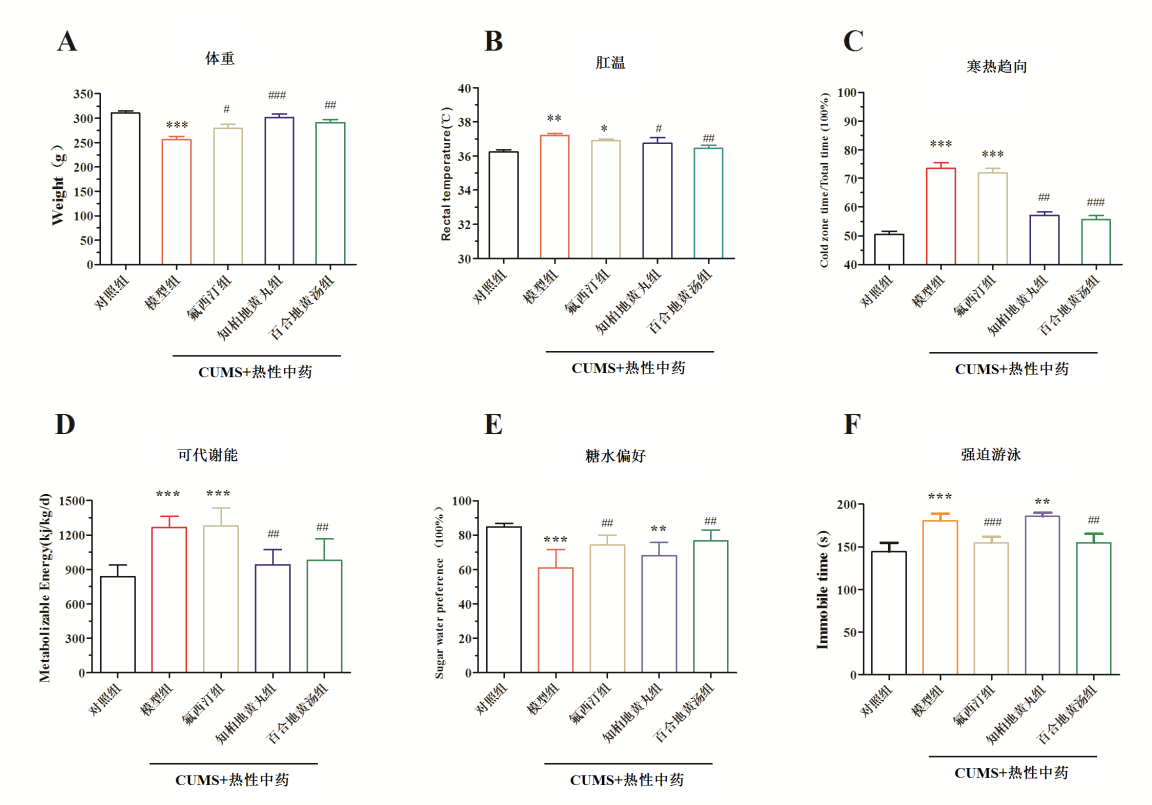

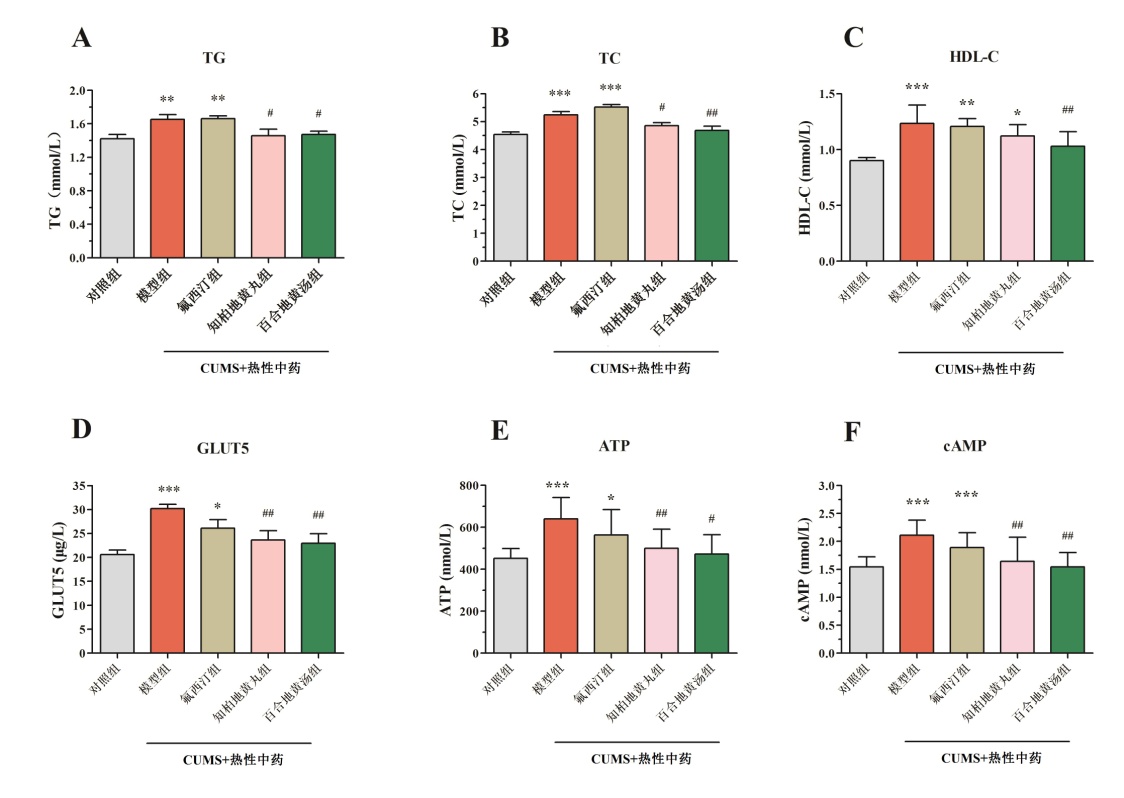
**

**
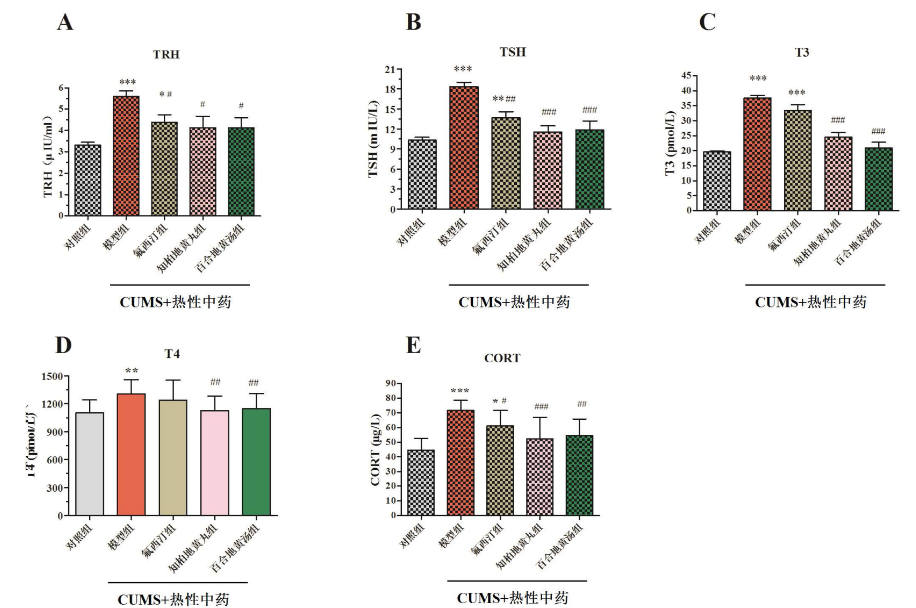
**

**
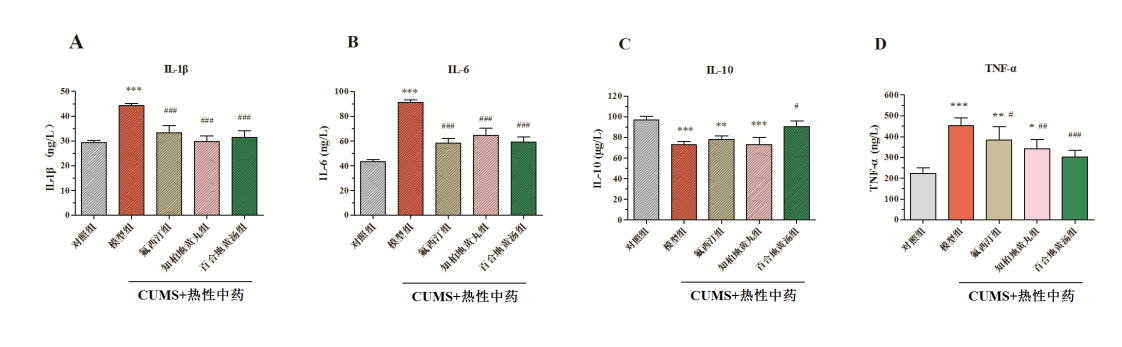
**

**
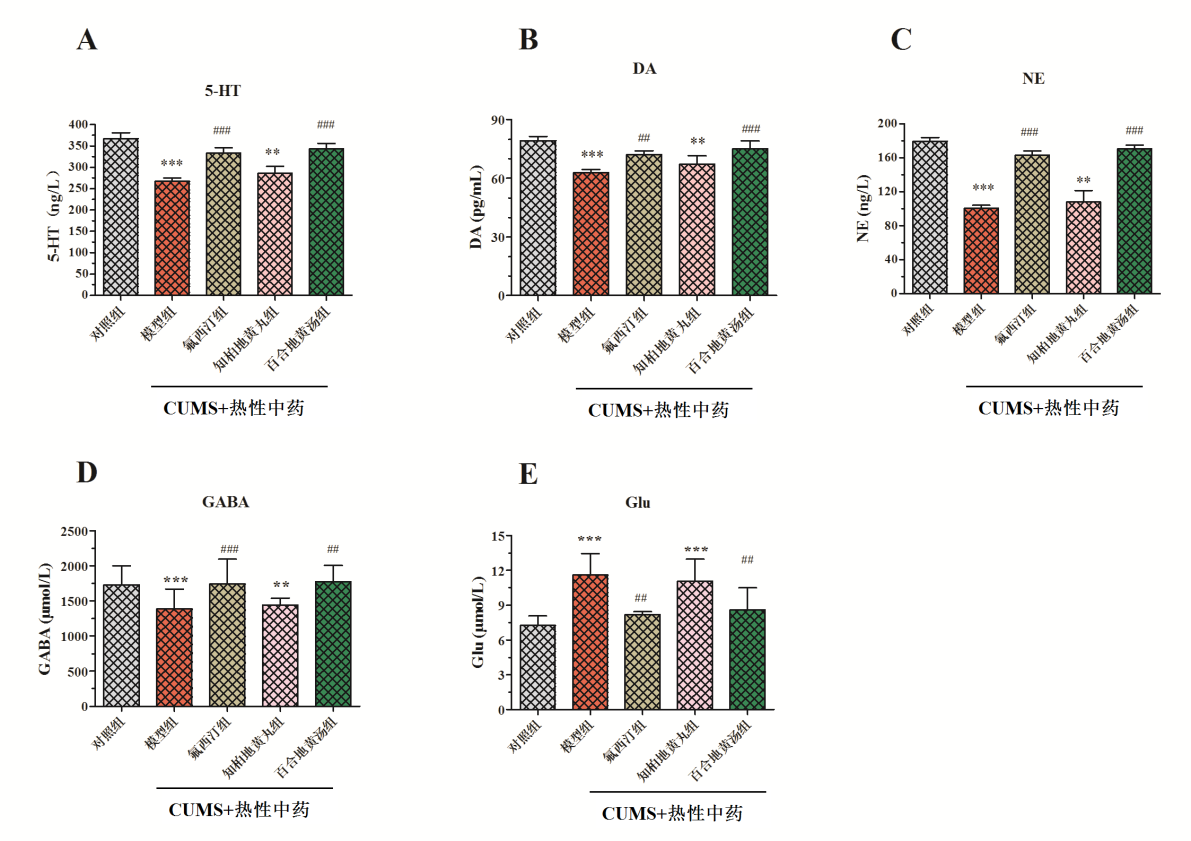
**
